# C9orf72-related amyotrophic lateral sclerosis-frontotemporal dementia and links to the DNA damage response: a systematic review

**DOI:** 10.3389/fnmol.2025.1671906

**Published:** 2025-11-18

**Authors:** Seham Almalki, Mohamed Salama, Matthew J. Taylor, Zubair Ahmed, Richard I. Tuxworth

**Affiliations:** 1 Department of Cancer and Genomic Sciences, School of Medical Sciences, College of Medicine and Health, University of Birmingham, Birmingham, United Kingdom; 2 Department of Biotechnology, Faculty of Science, Taif University, Taif, Saudi Arabia; 3 Department of Inflammation and Ageing, School of Infection, Inflammation and Immunology, University of Birmingham, Birmingham, United Kingdom; 4 Birmingham Centre for Neurogenetics, University of Birmingham, Birmingham, United Kingdom; 5 University Hospitals Birmingham NHS Foundation Trust, Birmingham, United Kingdom; 6 Centre for Trauma Sciences Research, University of Birmingham, Birmingham, United Kingdom

**Keywords:** ALS, FTD, ALS-FTD, C9orf72, DNA damage, DDR, DNA repair

## Abstract

The G4C2 repeat expansion in *C9orf72* is the most common genetic cause of amyotrophic lateral sclerosis (ALS) and frontotemporal dementia (FTD). While healthy individuals have fewer than 30 repeats, affected patients may carry hundreds to thousands. This expansion accounts for approximately 40% of familial ALS and 25% of familial FTD cases, and between 5 and 10% cases of sporadic ALS and FTD. Three overlapping pathological mechanisms have been proposed for the *C9orf72* expansion: loss of function due to protein deficiency, gain of function through RNA foci, and the production of toxic dipeptide repeat proteins (DPRs) via repeat-associated non-ATG (RAN) translation. This systematic review investigates the role of DNA damage in *C9orf72*-related ALS-FTD. Analysis of twelve peer-reviewed studies showed that *C9orf72* repeat expansions and DPRs compromise genome stability across four experimental models: human cell lines, induced pluripotent stem cell-derived neurons, rodent neurons, and postmortem tissue. We identified four mechanisms underlying DNA damage accumulation: disruption of the ATM pathway, impairment of DNA repair efficiency, formation of R-loops, and mitochondrial dysfunction with oxidative stress. In addition, several consequences of DNA damage were identified, including misrepair-mediated repeat expansion and activation of STING pathway. These findings highlight the key role of DNA damage in C9orf72-related pathology. Consistent with this, targeting DNA damage response factors extended lifespan and improved motor function in mouse models. This review highlights the contribution of DNA damage to C9orf72 pathology and suggest new therapeutic avenues, including personalized approaches based on genetic background.

## 1 Introduction

Amyotrophic lateral sclerosis (ALS) and frontotemporal dementia (FTD) are neurodegenerative disorders that exhibit significant overlap, sharing genetic, molecular, and pathological characteristics. ALS primarily affects motor neurons, leading to progressive degeneration, whereas FTD is characterized by atrophy of the frontal and temporal lobes (Balendra and Isaacs, 2018; Tang et al., 2020). The most common cause of inherited forms of ALS and FTD is a G4C2 repeat expansion in the first intron of the *C9orf72* gene (Balendra and Isaacs, 2018). Three overlapping mechanisms are proposed to contribute to C9orf72-related pathology: (Balendra and Isaacs, 2018) loss-of-function resulting from C9orf72 protein deficiency (Tang et al., 2020) RNA toxicity arising from bidirectionally transcribed repeat-containing RNA; and (Martin, 2008) production of dipeptide repeat proteins (DPRs), especially poly-GR and poly-PR *via* repeat-associated non-ATG (RAN) translation (Balendra and Isaacs, 2018; Tang et al., 2020).

Neurons are highly vulnerable to DNA damage and loss of genomic integrity due to several factors. These include their longevity, terminal differentiation status and their high metabolic activity. Various types of DNA damage occurs in neurons, driven primarily by reactive oxygen species and leading to oxidative lesions, single-strand breaks (SSB) (Martin, 2008) and the most harmful lesions – double-strand breaks (DSB) (Martin, 2008; Chatterjee and Walker, 2017). Increasing evidence shows that pathological samples from *C9orf72*-related ALS-FTD exhibit elevated levels of DNA damage markers, including *γ*-H2AX and phosphorylated ATM (pATM) (Walker et al., 2017; Farg et al., 2017). DNA damage repair mechanisms in post-mitotic neurons are limited. Two main mechanisms of repair are available: base excision repair (BER) for lesions that occur due to oxidative damage, and error prone non-homologous end-joining (NHEJ) for DSB lesions (Martin, 2008; Chatterjee and Walker, 2017). Growing evidence suggests that *C9orf72* expansions impair NHEJ by both disrupting assembly of the DNA-dependent protein kinase (DNA-PK) complex that binds to the ends of DSB to initiate repair and by inducing aberrant activation of DNA damage response (DDR) components such as γH2AX, p-ATM, 53BP1, and PARP-1, ultimately contributing to neurodegeneration (Farg et al., 2017).

Currently, Riluzole, Edaravone and a combination of Sodium Phenylbutyrate/Taurursodiol are the only FDA-approved pharmaceutical treatments for ALS, offering only modest slowing of disease progression and limited extension of patient lifespan (Tzeplaeff et al., 2023); there are no approved treatments for FTD. Tofersen, an antisense oligonucleotide therapy which targets *SOD1* mRNA (Tzeplaeff et al., 2023), is restricted to specific familial cases of ALS that make up only a very small proportion of ALS cases (Tzeplaeff et al., 2023), leaving the large majority of patients without effective therapies. This review aimed to systematically analyze available *in vitro* and *in vivo* studies to identify the molecular mechanisms underlying DNA damage accumulation and genomic instability in mammalian models of *C9orf72* ALS-FTD and from patient-derived cells and postmortem tissue. A better understanding of these mechanisms may reveal novel molecular targets that would improve the DDR and repair capacity of neurons to reduce genomic instability in ALS-FTD and thus offer new therapeutic strategies to protect against neurodegeneration.

## 2 Methods

### 2.1 Search strategy

This systematic review was part of larger review encompassing three of the best studied genetic causes of ALS-FTD: C9orf72, TDP-43 and FUS. The results were grouped into subsets according to genetic causes and prepared as separate articles [this review, plus (Almalki et al., 2025b; Almalki et al., 2025c)]. This systematic review was conducted using three databases: PubMed, EMBASE, and Web of Science, following the guidelines of the Preferred Reporting Items for Systematic Reviews and Meta-Analyses (PRISMA) (Page et al., 2021). Two authors (S. A. and Z. A.) conducted research in these databases using the same Boolean terms: ‘amyotrophic lateral sclerosis’ OR ‘ALS’ AND ‘DNA’ AND ‘double strand breaks.’ No date restrictions were applied, and the search extended to February 2025. The review focused on original research articles, excluding reviews, conference proceedings and included only publications in English. Studies of cell lines, mammalian models, patient-derived cell lines and studies using postmortem tissue were included. The full-text articles of all preliminarily selected studies were evaluated by S. A. to ensure they fit the criteria parameters and confirmed by Z. A.

### 2.2 Selection criteria

After removing duplicates, titles and abstracts were screened based on the following eligibility criteria: (1) the study used ALS or ALS-FTD associated genes; (2) the research focused, at least in part, on studying DNA damage, the DNA damage response (DDR), DNA repair pathway proteins, or manipulating genes or proteins associated with the DDR; and (3) studies were conducted in mammalian models or with patient-derived cells or tissue. Hence, studies using primarily non-mammalian models were excluded. Two studies on *C9orf72*-related ALS-FTD that used combinations of models: human induced pluripotent stem cell (iPSC) -derived neurons and *Drosophila* (Lopez-Gonzalez et al., 2019); and a mouse model, patient iPSC-derived neurons, and *Drosophila* (Maor-Nof et al., 2021), respectively. From these two studies, data related to the non-mammalian models, i.e., *Drosophila*, was excluded. Since inconsistencies in selection between the two authors (S. A. and ZA.) did not arise, resolution through discussion was not required. After the initial selection, we conducted a secondary screening of the bibliographies of included articles to identify additional relevant studies, which underwent the same selection process.

### 2.3 Data extraction

Data was extracted by S. A. from included studies using a pre-designed form. The following information was extracted from the selected studies: author, year, region, participants, methods, and whether the results pertained to DNA damage accumulation, changes in the efficiency of DDR or DNA repair pathways.

### 2.4 Risk of bias assessment

The selected articles were categorized into *in vitro* and *in vivo* studies, with *in vitro* studies encompassing research on cell lines, primary tissues, and iPSC cells derived from ALS or ALS-FTD patients. For these, we assessed the risk of bias using the Office of Health Assessment and Translation (OHAT) tool (Office of Health Assessment and Translation (OHAT) Division of the National Toxicology Program National Institute of Environmental Health Sciences, 2019), which consists of seven domains. The *in vivo* studies on live rodents were assessed using the SYRCLE risk of bias tool (Hooijmans et al., 2014), which comprises six domains, specifically tailored for animal-based studies. Two authors (S. A. and Z. A.) independently assessed all articles for risk of bias, and since there were no disagreements between the assessors, discussion to resolve any issues were not required.

### 2.5 Synthesis of results

Due to the high heterogeneity in the included studies and a paucity of numerical data, a meta-analysis of the pooled data was not possible. Hence, the synthesis of the data is presented in a narrative and tabular form.

## 3 Results

### 3.1 Study selection

**Figure 1:**
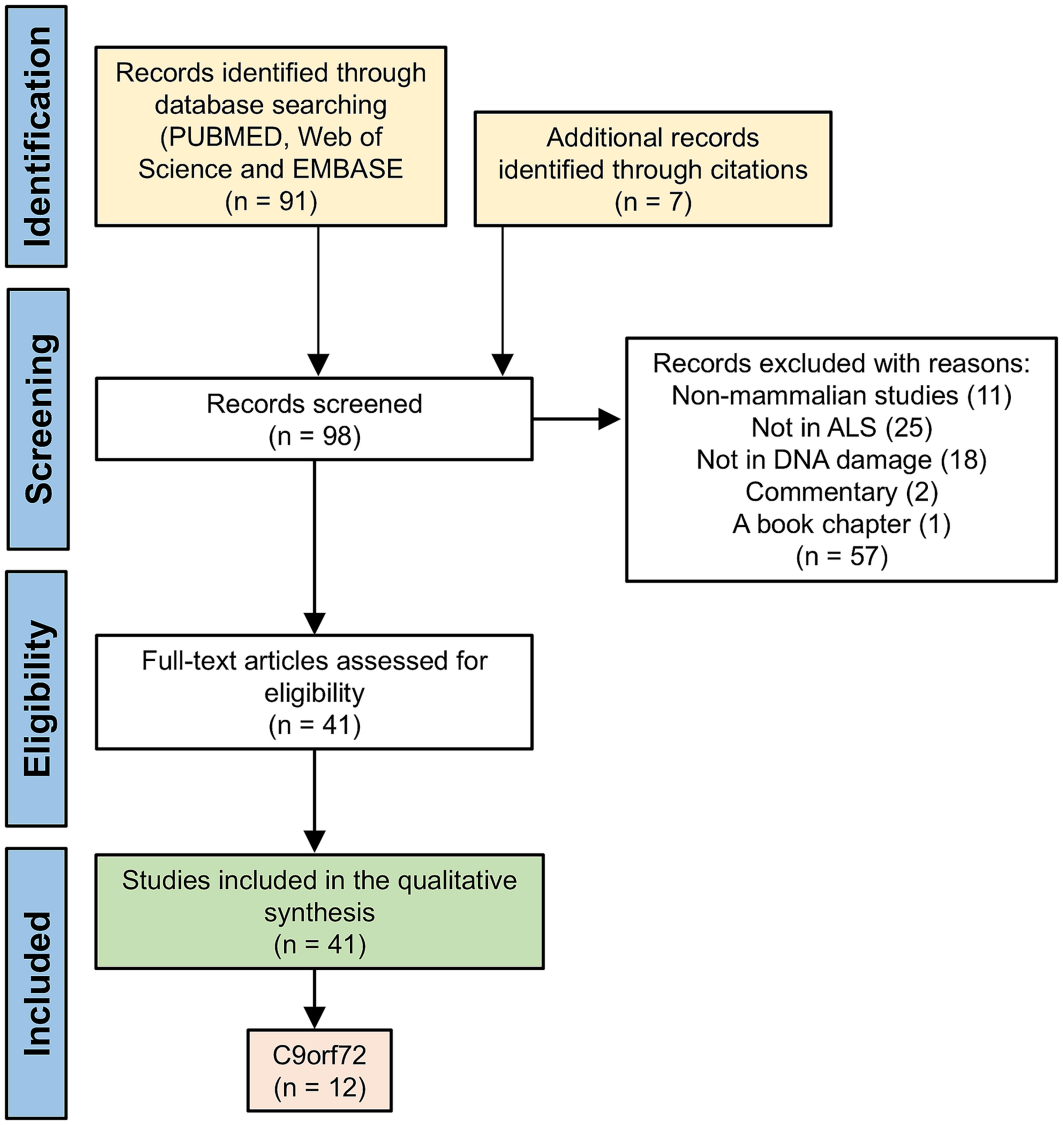
A PRISMA flow diagram illustrating the search and inclusion methods. 12 papers focusing on C9orf72-related ALS-FTD were met all inclusion criteria.

After removing duplicates, the search across the three databases identified a total of 91 studies (Figure 1). Through the review of bibliographies, we found an additional seven studies that were strongly linked to the review question, leading to a total of 98 studies included in our review. Following screening of the titles and abstracts, 41 studies remained eligible for full-text reading, and all were included in the qualitative synthesis. It is worth noting that these 41 studies covered a range of genes associated with ALS-FTD. Due to the large number of studies and results within each, a decision was made to analyze the findings in three separate reviews (see *Study characteristics* below) of which the 12 studies focusing on the *C9orf72* expansion are detailed in this review.

### 3.2 Study characteristics

The 41 peer-reviewed publications included were categorized into nine groups based on the ALS-FTD-associated genes they investigated: *C9orf72* expansion [12 studies: (Walker et al., 2017; Farg et al., 2017; Lopez-Gonzalez et al., 2019; Maor-Nof et al., 2021; Lopez-Gonzalez et al., 2016; Andrade et al., 2020; Nihei et al., 2020; Pal et al., 2021; He et al., 2023; Marques et al., 2024; Chang et al., 2024; Kojak et al., 2024)], TAR DNA-binding protein 43 [TDP-43; 12 studies: (Pal et al., 2021; Marques et al., 2024; Guerrero et al., 2019; Provasek et al., 2024; Tamaki et al., 2023; Gianini et al., 2020; Mitra et al., 2019; Nogami et al., 2022; Konopka et al., 2020; Mitra et al., 2025; Fang et al., 2023; Lee and Rio, 2024)] and Fused in Sarcoma (FUS) [12 studies: (Marques et al., 2024; Nogami et al., 2022; De Waard et al., 2010; Levone et al., 2021; Naumann et al., 2018; Jia et al., 2021; Kodavati et al., 2024; Niu et al., 2024; Deng et al., 2014; Gong et al., 2017; Wang et al., 2013; Wang et al., 2019)]. The *C9orf72* expansion features in this review (Almalki et al., 2025a), TDP-43 in Almalki et al. (2025b) and FUS in Almalki et al. (2025c). Notably, two of the *FUS* studies (Jia et al., 2021; Deng et al., 2014) also investigated related proteins belonging to the FET protein family: TATA-binding protein-associated factor 2 N (*TAF15*) and Ewing’s sarcoma (*EWS*). Additionally, a subset of studies investigated other ALS-FTD-related genes, including superoxide dismutase 1 (*SOD1*) (Kim et al., 2020; Mithal et al., 1999; Martin et al., 2007; Penndorf et al., 2017; Karanjawala et al., 2003), Senataxin (*SETX*) (Richard et al., 2020; Cohen et al., 2018), Heterogeneous nuclear ribonucleoprotein A1 (*hnRNPA1*) (Lee and Rio, 2024), and *Vps54* (Junghans et al., 2022) although each in only a small number of studies. These are described in Supplementary file 1.

### 3.3 Risk of bias assessment results

**Figure 2:**
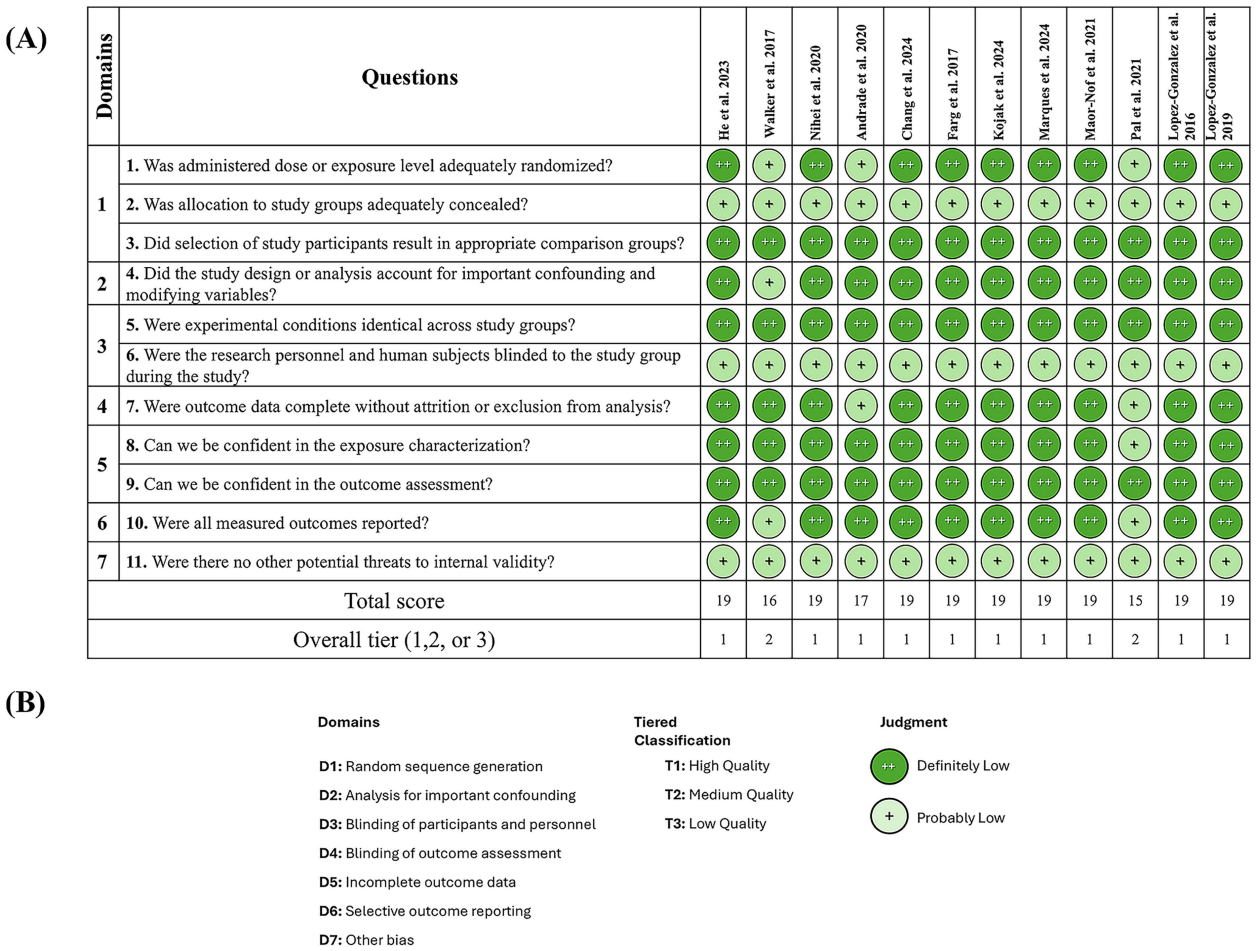
The OHAT tool for rating the risk of bias was used for the *in vitro* studies. **(A)** Risk of bias assessment across included studies. **(B)** Risk of bias domains and classification criteria.

Two tools were used to assess the risk of bias (RoB) in the 12 included studies focusing on *C9orf72* (Walker et al., 2017; Farg et al., 2017; Lopez-Gonzalez et al., 2019; Maor-Nof et al., 2021; Lopez-Gonzalez et al., 2016; Andrade et al., 2020; Nihei et al., 2020; Pal et al., 2021; He et al., 2023; Marques et al., 2024; Chang et al., 2024; Kojak et al., 2024). Since all 12 studies employed *in vitro* models, we evaluated them using the OHAT (Office of Health Assessment and Translation) tool (Office of Health Assessment and Translation (OHAT) Division of the National Toxicology Program National Institute of Environmental Health Sciences, 2019) (Figure 2). The assessment covered seven domains. Overall, 10 out of 12 studies (Farg et al., 2017; Lopez-Gonzalez et al., 2019; Maor-Nof et al., 2021; Lopez-Gonzalez et al., 2016; Andrade et al., 2020; Nihei et al., 2020; He et al., 2023; Marques et al., 2024; Chang et al., 2024; Kojak et al., 2024) were classified as Tier 1, indicating a low risk of bias, while two studies (Walker et al., 2017; Pal et al., 2021) were rated as Tier 2, reflecting a moderate RoB. This suggests generally strong methodological rigour. Domains 2 (important confounding factors) and 5 (incomplete outcome data) consistently showed high confidence across all studies, with nearly all cases rated as “++.”

**Figure 3:**
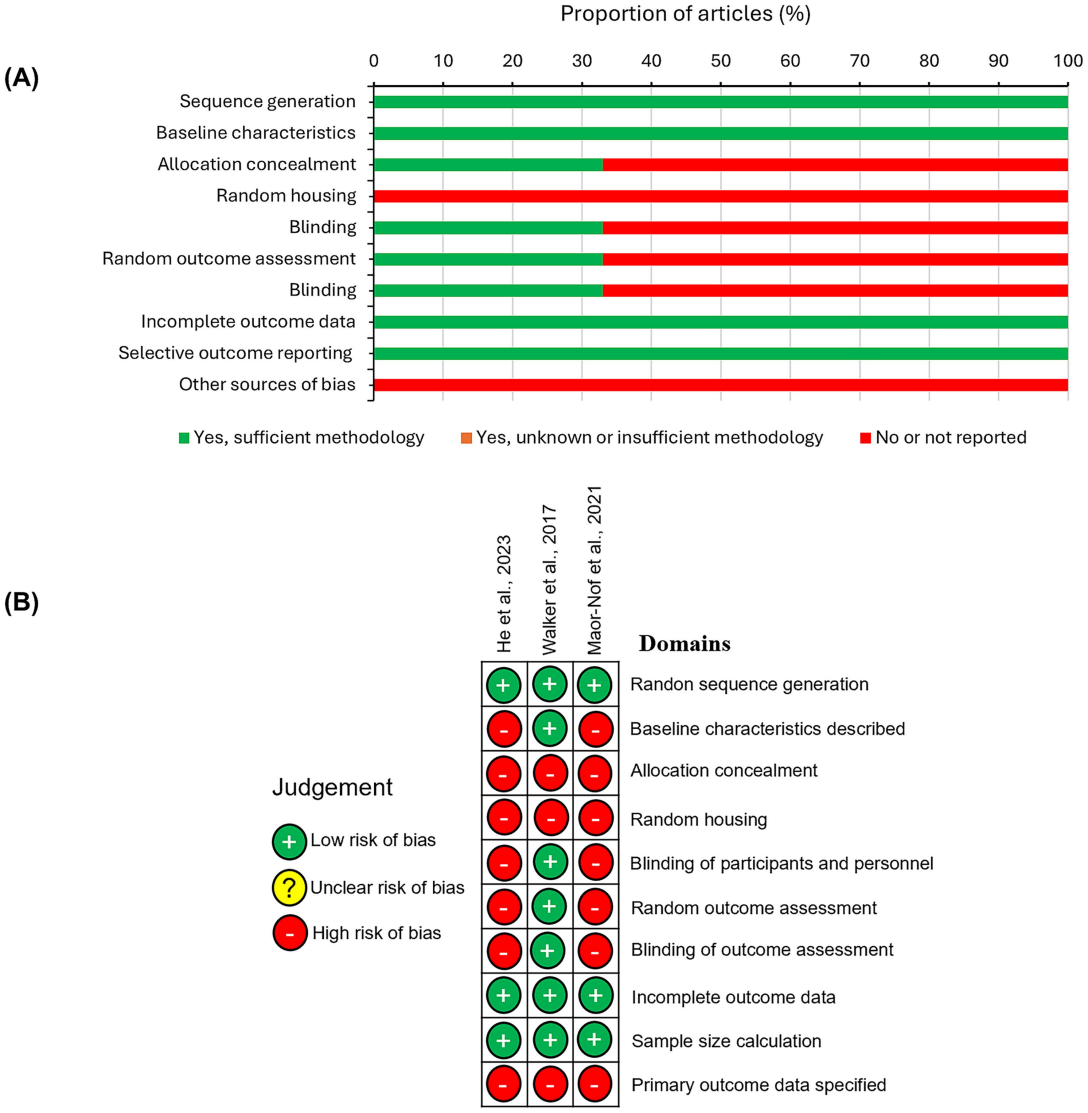
SYRCLE risk of bias (RoB) assessment for *in vivo* studies. **(A)** Percentage of the overall RoB for the three included *in vivo* studies. **(B)** The individual RoB summaries for the each study. Green (+): low risk; yellow (?): unclear risk; red (−): high risk.

The SYRCLE RoB tool (Hooijmans et al., 2014) was used to assess the three *in vivo* studies (Walker et al., 2017; Maor-Nof et al., 2021; He et al., 2023) (Figure 3). Overall, these studies demonstrated a high risk of bias. None reported on random housing or specified primary outcome domains. Additionally, two of the studies did not report allocation concealment, blinding of participants and personnel, random outcome assessment, or blinding of outcome assessment.

However, some domains showed no apparent risk of bias: each study adequately described random sequence generation, reported baseline characteristics, addressed incomplete outcome data, and performed sample size calculations.

In summary, while the *in vitro* studies exhibited an overall low risk of bias, the *in vivo* studies showed a high risk of bias, particularly in domains critical to the validity of their conclusions.

### 3.4 Results of studies reviewed

Of the 41 peer-reviewed publications, the *C9orf72* gene was investigated in 12 studies, encompassing both *in vitro* and *in vivo* methodologies (Table 1). The 12 studies investigated the link between DNA damage and C9orf72 expansion and pathology mechanisms. The studies were published between 2016 and 2024, covering four continents: Asia (He et al., 2023; Chang et al., 2024), Europe (Walker et al., 2017; Nihei et al., 2020; Pal et al., 2021), Australia (Farg et al., 2017), and North America. Most studies were conducted in the USA (Lopez-Gonzalez et al., 2019; Maor-Nof et al., 2021; Lopez-Gonzalez et al., 2016; Andrade et al., 2020; Marques et al., 2024; Kojak et al., 2024).

**Table 1:** Characteristics of the included studies.

Author/Year	Country	Model	Intervention	*C9orf72* mutations	Key mechanisms studied	Main findings	Method of detection
(A) Cell line-based studies							
Farg et al. (2017)	Australia	SH-SY5Y (h) (non-neuronal) Primary mouse cortical neurons	–	GR/ PR	DDR	Activation of DDR factors (γ-H2AX foci or p-ATM)	WB/IF/FISH
Walker et al. (2017)	UK	MRC5 (h)	Synthetic oligonucleotides cloned into expression vectors. ATM inhibitor	RRE GA	R-loops/DSBs/ DDR/ATM-mediated DNA repair	Elevated R-loops/DSBs/DDR reduction/Defective ATM-mediated DNA repair	WB/FISH-IF/ICC/Comet assay
Andrade et al. (2020)	USA	U2OS (h) (non-neuronal)	Poly-DPR DPR expression plasmids ETO	GA/GR/PR	DSB repair pathways/DPR interaction with NPM1	DPRs inhibit DSB repair pathways (NHEJ, SSA, and MMEJ)/Increased γH2AX signal	I-SceI based fluorescent reporter assays/IF/WB/ Repeat primed PCR
Nihei et al. (2020)	Germany	HeLa (h) (non-neuronal line)	Plasmid constructs DPRs	GR/ PR/GA	Poly-GA interactions with pATM	Unlike GR and PR, Poly-GA sequesters pATM in the cytoplasm, impairing DNA damage repair/Increased DNA damage	Co-IP/IF/WB
Maor-Nof et al. (2021)	USA	Primary cortical neurons (m)	p53 KO	PR	p53-mediated DNA damage response	p53 activation mediates DNA damage and apoptosis	WB/ICC/Live cell imaging
He et al. (2023)	China	U2OS (h) HEK293 (h) (non-neuronal lines) Primary cortical neurons (m)	CRISPR-Cas9/ siRNA DDR inhibitors ETP/PARP inhibitor	KO/KD KD/GR	DNA damage/DDR/NHEJ repair NEHJ repair, poly-GR-induced DNA damage	Colocalisation with γH2AX at DSB sites/ ETP significantly decreased γH2AX foci/Reduction in lig4/XRCC4 complex/Reduction in NHEJ efficiency KO and GR reduced NHEJ repair proteins (pDNA-PKcs, XRCC4, 53BP1)	H2AX, comet, NHEJ assays/live imaging IF/IB/comet assay/LA-PCR
Marques et al. (2024)	USA	Primary cortical neurons (m)	ETO/Glutamate	G4C2	Activation of STING pathway in response to DNA damage	G4C2 expansion causes DNA damage, Activating the STING pathway in neurons	IF
Chang et al. (2024)	Taiwan	Neuroblastoma N2a (m)	–	GR/PR	DNA damage induction by poly-GR/PR	GR and PR induce DNA damage (γH2AX)	H2AX assay
Kojak et al. (2024)	USA	*C9orf72*^hu96x/+^ mESCs (m)	H_2_O_2_/DSB/SSB introduction by CRISPR-Cas9	G4C2	G4C2 repeat instability mechanisms	Two mechanisms of repeat instability: Msh2-dependent minor expansions and DNA break-triggered major expansions	RP-PCR followed by capillary electrophoresis (CE)
(B) Patient iPSC derived neurons							
Lopez-Gonzalez et al. (2016)	USA	1- C9ORF72 ALS iPSCs-derived spinal MNs	–	–	Oxidative stress/ DNA damage	Increased oxidative stress and DNA damage	WB/IF/comet assay
		2- Control iPSC-derived MNs	Lentiviral vectors expressing poly-GA/GR	GR/GA			
Lopez-Gonzalez et al. (2019)	USA	C9ORF72 ALS/FTD patient and control iPSC-derived MNs	–	G4C2	NHEJ repair pathway pATM	Overactivation of Ku70, Ku80, DNA-PKcs, and pATM	qPCR/WB/IF
Andrade et al. (2020)	USA	C9orf72 ALS-derived MNs	–	–	DSB repair pathways	Increased DNA damage markers: γH2AX, Ku-70 (NHEJ), and pRAD52 (SSA)	WB
Nihei et al. (2020)	Germany	C9orf72 ALS-derived spinal MNs	RNAi-hnRNPA3	–	DSB factors (γH2AX and pATM)	Enhanced DSB factors in hnRNPA3 KD neurons	IF
Pal et al. (2021)	Germany	C9ORF72 ALS-derived iPSCs spinal MNs	–	HREs	DNA damage mechanisms	*C9orf72* mutations lead to DNA damage through both gain and loss of function mechanisms	IF
		healthy control iPSC-derived spinal MNs	–	–			
		CRISPR-edited isogenic spinal MN variants (C9-GC, C9-KO, WT-KO)	CRISPR/Cas9	Isogenic iPSC-derived spinal MNs (C9-GC, C9-KO, WT-KO)			
Maor-Nof et al. (2021)	USA	C9ORF72 ALS patient iPSC-derived MNs and CRISPR-corrected isogenic controls	CRISPR-Cas9 p53 KD (shRNA)	G4C2	p53-mediated DNA damage response	p53 activation mediates DNA damage and apoptosis	WB/ICC/Live cell imaging
He et al. (2023)	China	- NPC-derived neurons/C9orf72 ALS patient–derived NPCs	CRISPR-Cas9 H_2_O_2_/ETP ETP	KO	DNA damage/DDR/ NHEJ repair	Increased DNA damage/Reduction in γH2AX /Reduction in Lig4 and pDNA-PKcs	H2AX, comet, NHEJ assays
Marques et al. (2024)	USA	C9orf72 iPSC and controls differentiated into spinal MNs or cortical-like excitatory neurons	–	treatment with poly-(GR)20	Activation of STING pathway in response to DNA damage	Increased DNA damage (γH2AX)/activated STING pathway	IF/TR-qPCR
(C) Mouse-based studies							
Walker et al. (2017)	UK	Mouse brain sections	AAV9 viral vectors	GA/RRE	R-loops, DSBs, ATM-mediated DNA repair/HDAC4	Elevated R-loops, DSBs, and defective ATM-mediated DNA repair/ HDAC4 mislocalization	WB/IHC
Maor-Nof et al. (2021)	USA	AAV-tg mice	–	PR	p53-mediated DNA damage response	p53 activation mediates DNA damage and apoptosis	Lifespan assay/IF
Marques et al. (2024)	USA	Motor cortex (m)	–	G4C2	Activation of STING pathway in response to DNA damage	Increased DNA damage (γH2AX)/activated STING pathway	Immunoperoxidase staining
Kojak et al. (2024)	USA	Mouse model	–	G4C2	G4C2 repeat instability mechanisms	Two mechanisms of repeat instability: Msh2-dependent minor expansions and DNA break-triggered major expansions	Repeat-primed PCR/ capillary electrophoresis
(D) Human postmortem brain and SC tissues							
Farg et al. (2017)	Australia	SC section	–	–	Activation of DDR/DNA damage	Activation of DDR and increase DNA damage factors	IHC/WB/FISH/Co-IP
Walker et al. (2017)	UK	ALS patient SC sections	–	–	R-loop/DSBs/HDAC4	R-loop formation and defective ATM-mediated DNA repair/ increase nuclear HDAC4	IHC
Andrade et al. (2020)	USA	Patient brain tissues	–	–	DSB repair pathways	Overexpression of total RAD52	IF/WB
Nihei et al. (2020)	Germany	Patient brain tissues	–	–	Poly-GA sequestration of pATM	Poly-GA sequesters pATM	IHC
Marques et al. (2024)	USA	Motor cortex and SC tissues	–	–	Activation of STING pathway in response to DNA damage	Increased DNA damage (γH2AX)/activated STING pathway	IHC

Genes and proteins: C9orf72, chromosome 9 open reading frame 72; G4C2, hexanucleotide repeat expansion, RRE, repeat-associated non-AUG translation regulatory element, GA (glycine–alanine), GR (glycine-arginine), PR (proline-arginine), dipeptide repeat proteins (DPRs) generated from C9orf72 repeat expansions, Genetic tools and models: KD, knockdown; KO, knockout; AAV-tg, adeno-associated virus-transgenic mice. Experimental methods: CE, capillary electrophoresis; Co-IP, co-immunoprecipitation; IB, immunoblotting; ICC, immunocytochemistry; h, human; IF, immunofluorescence; IHC, immunohistochemistry; FISH, fluorescence in situ hybridization; LA-PCR, long-amplicon PCR; TR-qPCR, transcript-level quantitative PCR; RP-PCR, repeat-primed PCR; WB, western blot. DNA repair and damage assessment: Comet assay, single-cell DNA damage assay; γH2AX, phosphorylated histone H2AX (marker of DNA DSBs); 53BP1, p53-binding protein 1 (DDR marker); pDNA-PKcs, phosphorylated DNA-dependent protein kinase catalytic subunit; XRCC4, X-ray repair cross-complementing protein 4; NHEJ, non-homologous end joining; SSA, single-strand annealing; MMEJ, microhomology-mediated end joining; m, mouse; DSBs, double-strand breaks; SSBs, single-strand breaks; R-loops, RNA–DNA hybrids; DDR, DNA damage response. Chemical reagents: ETO, etoposide (topoisomerase II inhibitor); H₂O₂, hydrogen peroxide (oxidative stress inducer); CPT, camptothecin (topoisomerase I inhibitor); TBH, tert-butyl hydroperoxide (oxidative stress inducer); PARP inhibitor, poly(ADP-ribose) polymerase inhibitor; Glutamate, excitotoxic neurotransmitter; Antioxidant, compound *reducing oxidative damage. Cell and tissue types: NPCs, neural progenitor cells; MNs, motor neurons; mESCs, mouse embryonic stem cells; SC, spinal cord*.

Four distinct *C9orf72* expansion models were identified. Cell line-based studies were the most commonly employed [9 of 12; (Walker et al., 2017; Farg et al., 2017; Maor-Nof et al., 2021; Andrade et al., 2020; Nihei et al., 2020; He et al., 2023; Marques et al., 2024; Chang et al., 2024; Kojak et al., 2024)]. Patient iPSC-derived neuronal models were used in 8 of 12 studies (Lopez-Gonzalez et al., 2019; Maor-Nof et al., 2021; Lopez-Gonzalez et al., 2016; Andrade et al., 2020; Nihei et al., 2020; Pal et al., 2021; He et al., 2023; Chang et al., 2024), including neural progenitor cells (NPCs) (He et al., 2023), motor neurons (Lopez-Gonzalez et al., 2019; Maor-Nof et al., 2021; Lopez-Gonzalez et al., 2016; Andrade et al., 2020), and spinal motor neurons were each used in 4 studies (Lopez-Gonzalez et al., 2016; Nihei et al., 2020; Pal et al., 2021; Marques et al., 2024). Mouse models were employed in 4 of 12 studies (Walker et al., 2017; Maor-Nof et al., 2021; Marques et al., 2024; Kojak et al., 2024), and human post-mortem cortical and spinal cord tissue in 5 studies (Walker et al., 2017; Farg et al., 2017; Andrade et al., 2020; Nihei et al., 2020; Marques et al., 2024).

A number of outcomes were identified. The primary outcome involved DNA damage accumulation, including mechanisms and downstream responses. The secondary outcomes related to the effects of *C9orf72* pathology on DNA integrity, therapeutic modulation of mechanisms driving DNA damage accumulation, and behavioral studies.

### 3.5 Primary outcome: DNA damage accumulation: mechanisms and downstream responses

We identified four primary mechanisms contributing to the accumulation of DNA damage: (1) disruption of the ATM pathway; (2) disruption of DNA repair pathway efficiency; (3) formation of R-loops; and (4) mitochondrial dysfunction and oxidative stress. In addition, misrepair-mediated repeat expansion in C9orf72 and activation of the STING pathway, were observed as downstream responses to DNA damage and will be considered together in a fifth component. The following sections outline the key findings supporting each mechanism.

#### 3.5.1 Disruption of the ATM pathway

Walker et al. (2017) demonstrated that both *C9orf72* RNA repeat expansions (RRE; 10 or 102 G4C2 repeats) and poly-GA dipeptide repeats (DPR; 34 and 69 repeats) induced DNA damage, as shown by γH2AX immunostaining and comet assays. However, cells expressing either model exhibited an ATM signaling defect, evidenced by reduction in pATM and 53BP1 nuclear foci. The authors linked this defect to impaired RNF168-mediated ubiquitylation of histone H2A, a pathway normally required for 53BP1 recruitment and sustained ATM activity at DSBs. Importantly, overexpression of RNF168 or depletion of p62/SQSTM1 (a negative regulator of RNF168) restored 53BP1 and pATM foci in the *C9orf72* DPR model.

Transfection of MRC-5, a human fetal lung fibroblast cell line with either 10 or 102 G4C2 repeats or V5-epitope-tagged poly-GA (0, 34, or 69 DPR repeats) revealed length-dependent effects on 53BP1 and pATM signaling. While the 10-repeat RRE showed no significant change, the 102 RRE model exhibited a significant reduction in average 53BP1 (*p* < 0.001) and pATM foci (*p* < 0.05) per cell, compared to mock-transfected controls. Similarly, expression of poly-GA DPRs caused significant reductions in 53BP1 foci (34 DPRs: *p* < 0.001; 69 DPRs: *p* < 0.01) and pATM foci (34 DPRs: *p* < 0.001; 69 DPRs: *p* < 0.05), compared to mock-transfected models. These defects persisted even after induction of DNA damage with camptothecin (CPT) or tert-butyl hydroperoxide (TBH), as both RRE and DPR models maintained significantly fewer 53BP1 and pATM foci when compared with DMSO-treated controls. In rat cortical neurons, transfection with 102 G4C2 repeats or V5-epitope-tagged poly GA (0, 34, or 69 DPRs) followed by CPT treatment similarly reduced 53BP1 foci, although no significant effect on pATM was reported in this neuronal model.

The level of NBS1, a core component of the MRN complex responsible for activating ATM in response to DNA damage, was significantly increased in MRC-5 fibroblasts transfected with V5-epitope tagged poly GA (69 DPRs), compared to 0-V5 control (*p* < 0.01). This increase, together with elevated DSBs detected by γH2AX staining and comet assays, indicates that DNA damage was properly sensed despite the downstream ATM signaling defect.

Although no data were reported for the RRE model, MRC-5 fibroblasts co-transfected with poly GA DPRs (0 or 96 repeats, V5-epitope-tagged) and either RNF168-GFP or GFP alone showed a rescue of ATM signaling when RNF168 was overexpressed, with significant restoration of both 53BP1 (*p* < 0.001) and pATM foci (*p* < 0.006) compared to GFP controls. Similarly, siRNA-mediated-depletion of p62/SQSTM1 restored 53BP1 (*p* < 0.047) and pATM foci (*p* < 0.003) compared to normal p62 expression. These interventions also reduced DSBs (*p* < 0.029) and lowered R-loop accumulation (*p* < 0.037). This indicates that impairment in RNF168 function underlies the ATM defect in the *C9orf72* DPR model (Walker et al., 2017).

The defect in ATM signaling in *C9orf72* models was also associated with altered HDAC4 localization. Under normal conditions, HDAC4 is primarily cytosolic, and its localization is regulated by its phosphorylation status. In the context of ATM deficiency, hyperphosphorylation of HDAC4 by PP2A (a phosphatase negatively regulated by ATM) drives HDAC4 mislocalization to the nucleus. In rat cortical neurons, HDAC4 remained cytoplasmic in mock and 0-V5/GFP controls, but there was an approximately 3-fold increase in nuclear HDAC4 in 69-V5-epitope-tagged poly-GA DPRs. A similar effect was seen after treatment with the ATM inhibitor KU55933, indicating that an ATM-defect-linked nuclear HDAC4 mislocation (Walker et al., 2017).

Consistent results were observed *in vivo*. In cerebellar sections from mice injected with G4C2 repeats (10 or 102 RREs), or V5-poly-GA DPRs (0, or 69 repeats), nuclear HDAC4 accumulation was detected in 50% of Purkinje cells expressing 102 RREs compared to < 20% in the 10 RRE model. Likewise, 80% of Purkinje cells expressing 69-V5-poly-GA DPR displayed nuclear HDAC4 compared with 50% in the 0-V5 control. In spinal cord sections from patients with *C9orf72*-associated ALS, there was a significant increase in nuclear HDAC4 within motor neurons compared to control subjects (*p* = 0.0463) (Walker et al., 2017).

Nihei et al. (2020) reported that hnRNPA3 normally binds to sense (G4C2) and antisense (C4G2) repeats to suppress their accumulation. Loss or mislocalization of hnRNPA3 increased antisense RNA foci and DPRs, amongst which poly-GA sequesters pATM and impairs DSB repair. In fibroblasts from three patients and iPSC-derived MNs from two C9orf72 patients, the authors demonstrated that knockdown of hnRNPA3 (siRNA) significantly increased the number of antisense RNA foci. Immunostaining confirmed elevated RNA foci in Nucleolin-positive fibroblast nuclei and in TuJ1-positive neurons (*p* < 0.05), compared to siRNA controls. Extending this, *hnRNPA3* knockdown in HeLa cells transfected with sense or antisense repeats elevated levels of RNA foci and resulted in an increased production of antisense DPRs (poly-PA, PR, and GP). Together with their earlier findings that hnRNPA3 also regulates sense G4C2 repeat RNAs and DPRs, these results establish hnRNPA3 as a key suppressor of both sense and antisense C9orf72 repeat expression.

In HeLa cells, expression of GFP-tagged DPR (poly-GA, poly-GR, and poly-PR) differentially affected ATM signaling. Immunostaining for pATM revealed that poly-GR and poly-PR increased nuclear pATM foci (~1.5-2-fold compared to WT-GFP), whereas poly-GA significantly reduced nuclear pATM (<1-fold compared to WT-GFP). This reduction, observed both in WT and hnRNPA3 depletion cases, indicating that poly-GA sequesters pATM in the cytoplasm, which may impair pATM recruitment to DNA damage sites. Result from postmortem dentate gyrus granule cells aligned with the cell culture findings. In C9orf72 ALS-FTD patient tissue, poly-GA aggregates were frequently detected, and quantification showed that the percentage of granule cells with poly-GA inclusions was negatively correlated with the percentage of cells exhibiting nuclear pATM foci (*r* = −0.512, *p* < 0.05). Although, there was detectable nuclear pATM puncta in the immunostaining figure, their overall frequency was significantly decreased compared to controls.

#### 3.5.2 Disruption of DNA repair pathway efficiency

Andrade et al. (2020) reported that C9orf72 DPR directly impair DNA repair pathways, providing a potential mechanism for DNA damage accumulation in *C9orf72*-linked ALS-FTD disorders. To investigate this, four U2OS human osteosarcoma cell reporter lines were generated, each carrying a stably integrated GFP-based cassette to assess the efficiency of four DNA repair pathways: homologous recombination (HR), non-homologous end joining (NHEJ), microhomology-mediated end joining (MMEJ), and single-strand annealing (SSA). DNA breaks were induced by co-transfecting each reporter line together with an I-SceI endonuclease-encoding plasmid and a second plasmid encoding one of the DPRs (GA, GR, or PR) or as a control, no DPR. Repair efficiency was measured by the percentage of GFP-positive cells detected by flow cytometry. While none of the DPRs significantly affected HR repair, all three significantly impaired the NHEJ pathway: GA (−5% *p* < 0.05); GR (−8% *p* < 0.0005); and PR (−28% *p* < 0.0001), when compared to the empty vector. In addition, PR alone significantly reduced MMEJ efficiency (−23% *p* < 0.05), whereas both GA (−9% *p* < 0.01) and PR (−22% *p* < 0.0001) reduced SSA efficiency compared to the control.

Importantly, follow-up experiments focused only on NHEJ and SSA, where siRNA-mediated knockdown of Nucleophosmin (NPM1), a key protein in various DNA damage repair processes, further exacerbated the inhibitory effects of DPRs on NHEJ (*p* < 0.0001) and SSA (*p* < 0.0001), when compared to NPM1 siRNA or DPR-expression alone. This impairment is mediated, at least in part, through direct binding of DPRs to NPM1, as shown by co-localization with NPM1. Western blot analysis showed a significant increase in γH2AX level in U2OS cells transfected with HA-tagged PR DPRs in combination with NPM1 siRNA (pt < 0.0005), relative to a vector control. Super resolution STORM microscopy further revealed co-localization of γH2AX and pRAD52 in the nucleus of etoposide-treated cells indicating the activation of the SSA repair pathway in response to DNA damage (Andrade et al., 2020). Andrade et al. (2020) further demonstrated in *C9orf72* disease-relevant iPSC-derived MNs that an accumulation of the DNA damage marker γH2AX occurs together with elevated Ku70 and p-RAD52, suggesting dysregulation of the NHEJ and SSA pathways, respectively. These results are considered below under the secondary outcome: the effects of *C9orf72* pathology on DNA integrity.

He et al. (2023) demonstrated that in non-neuronal HEK293T cells, knockout (KO) of the *C9orf72* gene markedly reduced NHEJ efficiency, as shown by a significant decrease in GFP-positive cells following transfection with a cleaved NHEJ reporter construct compared to wild-type (WT) controls (*p* < 0.001). In contrast, alternative NHEJ (alt-NHEJ) and HR repair pathways remained comparable between KO and WT cells. He et al. (2023) further demonstrated that the C9orf72 protein associates with the DNA-PK complex following ETP treatment. In *C9orf72* KO HEK293T cells, pDNA-PKcs was significantly reduced 15 min after ETP treatment compared to WT cells (*p* < 0.05), although levels gradually increased over time. In parallel, co-immunoprecipitation experiments in WT HEK293T cells transfected with a *Flag-C9orf72* construct revealed that C9orf72 interacts with pDNA-PKcs, Ku70, and Ku80, and that this interaction is significantly enhanced following ETP treatment (*p* < 0.05). In contrast, after transfecting HEK293T cells with *HA-Ku70*, co-immunoprecipitation showed that Ku70/Ku80 interactions were significantly reduced in *C9orf72* KO cells (*p* < 0.01) compared to WT cells. The association of DNA-PKcs with the Ku70/Ku80 dimer was also reduced in the KO cells (*p* < 0.05), indicating that C9orf72 is important for the stabilization of the DNA-PK complex. Another key factor in the classical NHEJ (c-NHEJ) pathway is DNA ligase 4 (Lig4), the enzyme that seals broken DNA ends during DSB repair. Consistent with the defect observed when Ku70, Ku80 and DNA-PKcs function is impaired, live-cell imaging revealed a significant reduction in the maximum accumulation of GFP-Lig4 at the sites of laser-induced DSB in *C9orf72* KO cells (*p* < 0.001), similar to the effect of DNA-PKcs inhibition with NU7026 in WT HEK293T cells. Notably, recruitment kinetics were almost unchanged (WT τ1/2 = 1.18 ± 0.23 s versus KO τ1/ 2 = 1.22 ± 0.30 s). This could suggest that C9orf72 is required for efficient stabilization, but not the initial recruitment of Lig4 at DSB. Analysis of chromatin fractions showed that *C9orf72* KO cells exhibited a significant reduction in the intensity of Lig4/XRCC4 protein complex immunostaining following ETP treatment compared to WT HEK293T cells (*p* < 0.05), suggesting that C9orf72 promotes recruitment of the Lig4/XRCC4 complex to DNA damage sites. XRCC4 is the binding partner of Lig4 and together they form a complex that ensures efficient ligation of DNA ends during c-NHEJ. Consistent with cellular data, He et al. (2023) reported that brain sections from *C9orf72^−/−^* mice injected with AAV9-GFP-poly-GR (50 DPRs) displayed a significant reduction in pDNA-PKcs and XRCC4 foci per nucleus (*p* < 0.001) plus decreased 53BP1foci (*p* < 0.05), compared to WT mice injected with the same construct. Comet assays also revealed a significant increase in DNA breaks in *C9orf72^−/−^* mice injected with AAV9-GFP-poly-GR (*p* < 0.001).

#### 3.5.3 Formation of R-loops

Walker et al. (2017) reported increased R-loop accumulation in both RREs and poly-GA DPRs in two *C9orf72* models: non-neuronal MRC-5 fibroblasts and rat cortical neurons, as well as a significant increase in R-loop in spinal cord tissue from *C9orf72*-associated ALS patients. In MRC-5 cells, transfection with 102 G4C2 repeats significantly increase R-loops compared to both mock and 10 RRE transfections (*p* < 0.01) and, importantly, RNA foci colocalized with R-loops in the 102 RRE model. Moreover, R-loop accumulation was DPR-length dependent: ~ 4 R-loops were detected per cell with transfected with 34 DPRs (*p* = 0.046) but ~ 8 foci with 69 DPRs (*p* = 0.003), compared to controls.

Co-expression of *senataxin (SETX)*, an RNA/DNA helicase that resolves R-loops, significantly reduced R-loop formation, which in turn led to a reduction in DNA damage, as detected by γH2AX staining. In the 102 RRE model, <50% of cells with overexpressed *SETX* were γH2AX-positive compared to ~ 80% in controls cells with no *SETX* overexpression. In the DPR model, overexpression of *SETX* partially reduced DNA damage: in 69 DPR-expressing cells, γH2AX-positive nuclei decreased to ~ 60% (*p* < 0.05) when compared to cells with no overexpression of *SETX*, or to control cells expressing red fluorescent protein as a control. *SETX* overexpression also reduced cellular toxicity, as shown by decreased cleaved-PARP1 positivity (<5% vs. ~ 10% in 102 RREs) and (<5% vs. > 10% in 69 DPRs, *p* < 0.05) and reduced trypan blue-positive cells (<15% vs. > 30% in 69 DPRs). In rat cortical neurons, 102 RREs significantly increased formation of R-loops compared to mock and 10 RRE (*p* < 0.01). A similar effect was seen when longer DPRs were expressed in MRC5 fibroblasts (34 DPRs: *p* < 0.05; 69 DPRs: *p* < 0.01). Finally, in sections of spinal cord from ALS patients, motor neurons exhibited a significant increase in both R-loops (*p* < 0.0083) and DSBs (γH2AX, *p* < 0.0268) when compared to controls. In contrasting findings, Pal et al. (2021) reported no increase in R-loop formation across iPSC-derived spinal MNs carrying either *C9orf72* knockout or a G4C2 repeat expansion, despite a clear increase in DSBs in these cells.

#### 3.5.4 Mitochondrial dysfunction and oxidative stress

One study by Lopez-Gonzalez et al. (2016), reported an age-dependent increase in reactive oxygen species (ROS) levels in *C9orf72* patient iPSC-derived motor neuron cultures, indicating increased oxidative stress on the cells. This was accompanied by increased DNA damage. The increase in ROS levels was significantly elevated in *C9orf72* patient iPSC-derived motor neurons after 8 weeks in culture and continued to rise at 3–4 months (*p* < 0.01) compared with control iPSC-derived motor neurons. The rise in ROS levels in mitochondria was specific to poly-GR expression and was not observed with poly-GA. Using the MitoSOX assay, 2-week-old control iPSC-derived motor neurons transduced with poly-GR (80 repeats) showed an increase in ROS (*p* < 0.01) compared to motor neurons transduced with an empty vector, whereas poly-GA (80 repeats) had no effect on mitochondrial ROS levels.

Additionally, both 4 month-old *C9orf72* patient or healthy control iPSC-derived MNs transduced with poly-GR (80 repeats) showed a significant increase in mitochondrial membrane potential (*p* < 0.001), as assessed by TMRM staining and flow cytometry, compared with their respective control transduction experiments with an empty vector. In parallel, interactome analysis in HEK293T cells expressing 80 repeats of poly-GR-GFP or GFP alone identified a strong enrichment of mitochondrial ribosomal proteins interacting with poly-GR: 47% of mitochondrial ribosomal protein interacted with the poly-GR *vs.* 20% of cytoplasmic ribosomal proteins. This preferential binding to mitochondrial ribosomal proteins provides one mechanistic explanation for the mitochondrial dysfunction observed in patient iPSC-derived MNs, which is likely to contribute to increased oxidative stress on cells and a consequent increase in DNA damage.

#### 3.5.5 Downstream responses to DNA damage: mismatch repair expansion

Kojak et al. (2024) demonstrated that DNA damage including SSBs and DSBs directly triggers expansion of the *C9orf72* G4C2 repeats. The study showed that these expansions are mediated by specific DNA repair pathways: the DNA mismatch repair (MMR) pathway, driven by Msh2, which promotes continuous small-scale expansions, and the homology-directed repair (HDR) pathway, driven by Rad51, which mediates large-scale expansions following DNA strand breaks. Kojak et al. (2024) generated humanized *C9orf72* alleles and inserted them into mouse embryonic stem (mES) cells through homologous recombination, replacing the corresponding mouse genomic region with the human counterpart containing G4C2 repeats, yielding alleles with 3, 31, or 96 repeats. In cultured mES cells, exposure to the global DNA-damaging agent H_2_O_2_ resulted in an average increase of nine G4C2 repeat compared to seven repeats in vehicle-treated cells.

CRISPR/Cas9 was used to induce DSB near the *C9orf72* expansion in the same mES cell model before the cells were subcloned. 18.5% of clones lacked PCR products when amplification was attempted with *C9orf72* gene-specific primers, consistent with both deletions or alterations. However, among the 81.5% of clones that yielded PCR products, 18.3% displayed significant repeat expansions (>10 additional G4C2 repeats), 42.7% retained a repeat length similar to the parental 96x repeat allele, and 21.8% displayed contractions (<90 repeats). These results were confirmed using two-primer gene-specific PCR and three-primer repeat-primed PCR (RP-PCR) with capillary electrophoresis. Inducing breaks 5` or 3` of the repeat tract provide comparable frequencies of expansions and contractions.

When DSB were generated, expansions, contractions, and deletions resulted in both 96x and 31x repeat lines, with expansions more frequent in the 96x repeat line (3.4%) and absent in the 31x repeat line. In contrast, inducing SSB in the 96x line resulted in expansions without large deletions, with expansions 4.85x more frequent than contractions. However, large expansions (>10 additional G4C2 repeats) still occurred after a 5` DSB was generated even when *Msh2* was knocked out. This indicates that MMR contributes to baseline instability but it is not required for break-induced expansions.

#### 3.5.6 Consequences of DNA damage: activation of the STING pathway

In our systematic review, Marques et al. (2024) was the only study included that investigated activation of the STING pathway as a downstream response to DNA damage. Three *C9orf72* models were studied: human postmortem tissue, a mouse model in which an AAV was used to express G4C2_149_ in the brain, and human iPSC-derived neurons. In addition, primary mouse cortical neurons from WT embryonic mice were used to ask whether neurons have functional STING pathway. The study investigated the activation of STING pathway *via* both canonical and non-canonical pathways. The canonical STING pathway is activated when the cytosolic DNA sensor, cGAS, detects DSBs, producing cGAMP that activates STING. Two main factors are recruited in this pathway which together drive the production of type 1 interferon: the kinase TBK1 and transcription factor IRF3. On the other hand, the non-canonical STING pathway functions independently of cGAS and can lead to different downstream effects, including the activation of the NF-κB pathway which can activate alternative gene programs and even inhibit the canonical response.

In primary mouse cortical neurons cultured at embryonic day 13–14, treatment with the DNA-damaging agent etoposide induced a significant increase in both nuclear γH2AX and cytoplasmic STING areas, compared to vehicle-treated neurons (*p* < 0.001). Treating the neurons with extracellular glutamate that induces excitotoxicity and results in mitochondrial dysfunction and increased ROS production and oxidative stress had a similar outcome. In parallel, both agents also increased the nuclear localization of p-IRF3 and p-NF-κB (*p* < 0.0001) compared to vehicle-treated neurons, indicating activation of both the canonical (IRF3) and non-canonical (NF-κB) STING pathways in response to DNA damage.

In human postmortem sections from eight *C9orf72* cases (five ALS, and three ALS-FTD), immunohistochemistry revealed strong STING activation in layer V Betz cells, and ventral spinal cord SMNs, the most vulnerable neuronal populations. In contrast, no activation was observed in layer II/III neurons or the occipital cortex. Quantification showed a ~ 5-fold increase in STING-positive neurons compared to eight p-TDP-43-negative Alzheimer’s disease sections and six non-neurological disease controls (*p* < 0.001).

In one-year old *C9orf72* AAV (G4C2)_149_ mice, co-immunostaining exhibited a significant accumulation of STING signal specifically in layer V CTIP2 + cortical motor neurons (18.9%), but not in layer II/III neurons when compared to the control *C9orf72* AAV (G4C2)_2_ mice. Consistent with this, there was an increase in p-IRF3 and p-NF-κB staining in the *C9orf72* AAV (G4C2)_149_ cortices but not in the (G4C2)_2_ cortices, indicating activation of both the canonical (IRF3) and noncanonical (NF-κB) STING pathways, although no statistical values were provided for these markers.

Finally, in iPSC-derived neurons, both 35-day spinal motor neurons (SMNs) and 10-day Neurogenin-2-induced cortical-like neurons from five *C9orf72*-related ALS patients exhibited a time-dependent increase in the intensity of canonical (IRF3) and noncanonical (NF-κB) STING pathway factors compared to their corresponding controls (*p* < 0.001 and *p* < 0.0001, respectively). At the transcript level, mRNA expression of canonical and noncanonical pathway genes was also significantly elevated (SMNs: *p* < 0.05 and NGN2 neurons: *p* < 0.001) compared to their controls. Treating the neurons derived from healthy control patients with extracellularly applied poly-GR 20xDPRs for 24 h resulted in a significant increase in both γH2Ax-positive neurons and STING signal (*p* < 0.0001) relative to neurons treated with either poly-GA 10xDPRs or with a vehicle control.

### 3.6 Secondary outcomes

We identified six secondary outcomes that will be discussed in turn: (1) effects of C9orf72 pathology on DNA integrity; (2) DNA-level effects of G4C2 repeats; (3) outcomes related to RNA repeat expansions; (4) outcomes related to DRPs; (5) therapeutic modulation of the mechanisms of DNA damage accumulation; and (6) findings of behavioral studies. However, even within the DNA-level effects, RNA- or DPR-mediated effects cannot be entirely ruled out and therefore, the distinction between these categories are not always clear cut.

#### 3.6.1 Effects of C9orf72 loss of function on DNA integrity

He et al. (2023) demonstrated that *C9orf72* is rapidly recruited to DSB sites. In U2OS cells transfected with *GFP-C9orf72*, laser micro-irradiation induced immediate accumulation of C9orf72 protein at DNA lesions (τ1/2 = 0.19 ± 0.06 s), co-localizing with the DSB marker, γH2AX. Upon treatment with the ETP, the level of C9orf72 in the chromatin fraction was increased in HEK293T cells and in mouse primary cortical neurons (*p* < 0.05), as shown by immunoblotting analysis. Two studies (Pal et al., 2021; He et al., 2023) investigated loss of function of *C9orf72* and its link to DNA integrity. Pal et al. (2021) reported that knockout of *C9orf72* in wild-type iPSC-derived spinal motor neurons led to a significant increase in γH2AX foci at day 80 when compared to WT control (*p* < 0.001), with DSBs accumulating in MAP2-positive neurons. In contrast, 53BP1 staining did not reveal significant differences between *C9orf72* knockout and control motor neurons. He et al. (2023) demonstrated that knockout of *C9orf72* impairs the DDR despite increased levels of DNA breaks. Using CRISPR to generate a *C9orf72* KO in iPSC-derived neuronal progenitor cells (NPCs) and in mouse cortical neurons, *C9orf72*-deficient neurons exhibited significantly longer comet assay tails compared to controls, both at baseline and after treatment with H_2_O_2_, indicating an accumulation of DNA damage. However, immunostaining revealed a significant reduction in γH2AX intensity (*p* < 0.001) and DNA-PKcs phosphorylation (*p* < 0.001) in *C9orf72*-deficient cells following ETP treatment, when compared to controls, indicating defective DDR signaling and impaired NHEJ repair.

#### 3.6.2 DNA-level effect of G4C2 repeats

Eight studies (Lopez-Gonzalez et al., 2019; Maor-Nof et al., 2021; Lopez-Gonzalez et al., 2016; Andrade et al., 2020; Nihei et al., 2020; Pal et al., 2021; He et al., 2023; Marques et al., 2024) used iPSC-derived neuronal systems to investigate the consequences of repetitive protein sequence expansion on *C9orf72*-mediated ALS-FTD. Among them, Maor-Nof et al. (2021) mainly focused on the neuroprotective effects of p53 knockdown, which will be discussed in detail below, while Marques et al. (2024) primarily addressed neuronal STING pathway activation, already covered above. The remaining six studies specifically examined the impact of the *C9orf72* G4C2 repeat expansion in different iPSc-derived cell types, including neuronal progenitor cells (NPCs) (He et al., 2023), motor neurons (Lopez-Gonzalez et al., 2019; Andrade et al., 2020; Nihei et al., 2020), and spinal MNs (Lopez-Gonzalez et al., 2016; Nihei et al., 2020; Pal et al., 2021).

He et al. (2023) reported that *C9orf72* ALS patient-derived NPCs exhibited a significant increase in DNA damage, as indicated by longer comet assay tails even after 24 h recovery period following treatment with ETP compared to healthy controls (*p* < 0.05). Despite this elevated levels of DNA damage, γH2AX was significantly reduced. Immunostaining after 30 min of ETP treatment showed significantly lower γH2AX intensity in *C9orf72* ALS NPCs relative to controls (*p* < 0.01). A significant reduction was also observed in pDNA-PKcs and Lig4 factors (*p* < 0.01).

Three studies (Lopez-Gonzalez et al., 2019; Andrade et al., 2020; Nihei et al., 2020) provides evidence that levels of DNA repair factors increase with neuronal age in *C9orf72*-related iPSC-derived MNs, involving factors from both the NHEJ and SSA repair pathways. Lopez-Gonzalez et al. (2019) showed that three-month-old *C9orf72* MNs from four patients had a significantly elevated levels of Ku80 mRNA (*p* < 0.0015) plus in both Ku70 (*p* < 0.0001) and Ku80 (*p* < 0.0002) protein levels, when compared to controls. In contrast, 2 week-old MNs showed no such increase in the protein levels. Immunostaining confirmed that Ku80 signal localized specifically to ChAT-positive neurons but not to GFAP-positive astrocytes. In parallel, protein lysates from three-month-old *C9orf72* MNs also displayed increased PKcs and p-ATM compared to controls (*p* < 0.0002). Andrade et al. (2020) analyzed 60-day-old MNs derived from two *C9orf72* ALS patients and reported significant increase in γH2AX protein in both lines (*p* < 0.0005 and *p* < 0.0005, respectively) compared to controls. However, only one patient line showed significant increase in Ku70 and pRAD52 levels (*p* < 0.0001), whereas MNs from patient line 2 did not differ from controls. This is attributed to differences in *C9orf72* promotor methylation, which led to reduced expression of the repeat expansion and, in turn a reduced activation of DDR pathways. Nihei et al. (2020) reported that normally hnRNPA3 binds both sense and antisense RNA repeats and prevents dipeptide repeat formation. However, cytoplasmic mislocalization of hnRNPA3 resulting in nuclear depletion enhances the production of DPRs. Consistent with this, siRNA-mediated *hnRNPA3* knockdown in *C9orf72* patient-derived iPSC neurons significantly enhanced both γH2AX and p-ATM foci compared to controls (*p* < 0.01).

Two studies (Lopez-Gonzalez et al., 2016; Pal et al., 2021) investigated change in DNA damage and DDR signaling in *C9orf72* patient iPSC-derived spinal MNs (SMNs). Lopez-Gonzalez et al. (2016) demonstrated a significant increase in DNA damage in four patient-derived SMN lines using comet assays at 4 months of culture. Western blotting revealed elevated γH2AX protein in patient SMNs, with one line showing a strong signal although no quantification data was provided. Immunostaining confirmed accumulation of γH2AX foci specifically in ChAT-positive SMNs but not in astrocytes. All three assays showed that DNA damage was first detectable at around 8 weeks of culture and increased at 3–4 months. In parallel, several DDR factors were elevated in *C9orf72* SMNs, including p53, p-p53, ATM, and GADD45. Pal et al. (2021) examined iPSC-derived SMNs from five ALS patients. At day 21 of differentiation, there was no increase in γH2AX signal, however, patient SMNs at day 80 showed an elevation in γH2AX foci, but not in 53BP1 compared to controls. The γH2AX signal was present in MAP2-positive neurons but absent from glia. Importantly, longitudinal analysis identified that axonal trafficking defects appeared by day 40, however, DNA damage accumulated only after day 60, which suggests that DNA breaks may occur downstream of trafficking defects. Similarly, isogenic C9-KO SMNs in which the intronic G4C2 expansion is retained but the protein-coding exon 2 was deleted exhibited accumulation of both γH2AX and 53BP1 foci at day 80 (*p* < 0.001), when compared to control, while, C9 gene corrected (C9-GC) SMNs, in which the pathogenic intronic G4C2 expansion was excised by CRISPR/Cas9 and replaced with the WT repeat with 1–3 repeats, exhibited an increase in neither. In parallel, cleaved caspase-3, a marker of apoptosis, was significantly elevated in the C9-KO SMNs at day 80, but not in controls or in the corrected SMNs.

In the ventral horn region of lumbar spinal cord tissue from *C9orf72*-ALS Caucasian patients, Farg et al. (2017) reported that approximately 80% of vCHAT positive MNs from C9orf72 patients expressed p-ATM (Ser1981), compared to about 40% in controls (*p* < 0.05). Western blot analysis from the same tissue further revealed a significant increase in cleaved PARP-1 (84% increase; *p* < 0.0001) and 53BP1 (70% increase; *p* < 0.001) *C9orf72*-ALS patients compared to controls. Additionally, Andrade et al. (2020) demonstrated elevated level of RAD52 expression in human post-mortem brain tissue. The study measured DNA repair proteins across three regions: motor cortex, occipital cortex, and cerebellum. All three regions exhibited increased total RAD52 in *C9orf72* ALS-FTD samples compared to sporadic ALS (sALS) and unaffected controls (*p* = 0.035, and *p* = 0.004), respectively. However, considerable variability was observed within groups: in the motor cortex, two of six *C9orf72* samples exhibited RAD52 levels lower than in unaffected controls and in samples from sALS. In the cerebellum, one sample exhibited levels similar to unaffected or sALS cases. The occipital cortex provided the most consistent signal, with significantly elevated RAD52 level compared to both sALS (*p* = 0.0119) and unaffected controls (*p* = 0.0023). By contrast, pRAD52 and 53BP1 protein levels were highly variable across samples, and no significant differences were detected between diagnostic groups in any brain region, as confirmed by western blot analysis. Additionally, Nihei et al. (2020) reported a relationship between nuclear hnRNPA3 levels and DNA damage in postmortem frontal cortex from *C9orf72* ALS-FTD cases. Immunostaining showed that patients with low nuclear hnRNPA3 exhibited minimal DNA damage. Quantification confirmed a significant negative correlation between nuclear hnPNP3 intensity and γH2AX positivity (*r* = −0.520, *p* < 0.05), which suggests a link between hnRNPA3 loss in patient neurons and accumulation of DSBs.

#### 3.6.3 RNA repeat expansions (RREs)

One study investigated the impact of RNA repeat expansion (RREs) on DNA damage accumulation using two models: non-neuronal immortalized cell lines plus spinal cord tissues from *C9orf72*-ALS patients. Walker et al. (2017) reported a significant increase in γH2AX foci in MRC-5 fibroblasts expressing 102 RRE compared to 10 RRE and mock-transfected cells (*p* < 0.001). In addition, a neutral comet assay using DNA extracts from HEK293T cells transfected with 102 RREs showed a significant increase in comet tail length compared to 10 RRE and mock-transfected cells (*p* < 0.01). Consistent with this, in spinal cord tissue from ALS patients, motor neurons exhibited a significant increase in γH2AX compared to controls (*p* = 0.0268). Another study by Farg et al. (2017) examined lumbar spinal cord tissue from *C9orf72*-ALS Caucasian patients and found a significant increase in γH2AX foci within SM132-positive MNs. Quantification of γH2AX protein level showed a 30.6% increase in five patient samples (*p* < 0.001) compared to controls, although no increase was seen in another two patient samples. Consistent, γH2AX nuclear foci colocalized with *C9orf72* RNA foci detected by FISH, supporting an association between repeat RNA and DNA damage.

#### 3.6.4 C9orf72 dipeptide repeats

Walker et al. (2017) reported an increase in DNA damage in cultured cells expressing poly-GA DPRs. In MRC-5 fibroblasts expressing 34 or 69-V5-tagged poly-GA DPRs, elevated levels of γH2AX foci were observed compared with 0-V5 controls (*p* < 0.01 and *p* < 0.001, respectively). Similarly, a neutral comet assay in HEK293T cells transfected with poly-GA DPRs showed a significant increase in comet tail length in 34 and 69-V5-tagged poly-GA DPR-expressing cells compared to controls (*p* < 0.001). In addition, there were observed defects in ATM activation and 53BP1 recruitment, as described above. Using codon-altered FLAG-tagged constructs to express poly-GR or poly-PR (100 DPRs), Farg et al. (2017) reported a significant increase in γH2AX immunostaining in undifferentiated SH-SY5Y neuroblastoma cells (*p* < 0.001) and in primary mouse cortical neurons (GR: *p* < 0.01 and PR: *p* < 0.001), compared to empty vector. In the SH-SY5Y cells, there was also a significant increase in p-ATM foci (*p* < 0.05). Additionally, Nihei et al. (2020) reported that expression of GFP-tagged DPR (poly-GA, poly-GR, and poly-PR) in HeLa cells significantly increased DSB accumulation, as indicated by nuclear γH2AX foci. This effect was further exacerbated by hnRNPA3 depletion (A3KO). Quantification showed that the strongest increase for γH2AX was seen with expression of poly-GA (4-fold), followed by poly-PR (3.3-fold) and poly-GR (3-fold) when compared to controls. Subcellular distribution of the DPRs also differed: poly-GA and poly-GR GFP signal were mainly cytoplasmic, whereas poly-PR was present in both the nucleus and cytoplasm.

Pal et al. (2021) examined DNA damage in iPSC-derived SMNs from five ALS patients carrying *C9orf72* HREs. After 21 days of differentiation, γH2AX and 53BP1 foci were comparable to controls but by day 80 the patient-derived neurons showed a significant elevation in γH2AX (*p* < 0.001) and 53BP1 (*p* < 0.001) foci, coinciding with poly-GP and poly-GA accumulation. γH2AX foci were quantified in MAP2-positive neurons, whereas 53BP1 foci were analyzed in Hoechst-positive nuclei. Neurons displaying poly-GP inclusions in neurites or poly-GA inclusions in a perinuclear location also frequently contained nuclear γH2AX or 53BP1 foci, respectively. This may indicate co-occurrence of DPR accumulation with DNA damage. Moreover, there was direct colocalization between γH2AX or 53BP1 themselves.

Chang et al. (2024) reported an increase in DNA damage as measured by γH2AX, in mouse neuroblastoma N2a cells treated with synthesized poly-GR DPRs (30 repeats) or poly-PR DPRs (30 repeats). Both DPRs elevated γH2AX foci counts in the nuclei of the cells, but the effect was stronger with poly-GR DPRs. Specifically, poly-GR DPRs significantly increased γH2AX foci number, area and intensity (*p* < 0.0001) compared to poly-GR DPRs (10 repeats) and BPS treatment. In contrast, poly-PR DPRs (30 repeats) increased γH2AX foci number but did not significantly alter foci area or intensity relative to PBS.

#### 3.6.5 Therapeutic modulation of mechanisms driving DNA damage

One strategy aimed at reducing DNA damage by targeting oxidative stress was suggested by Lopez-Gonzalez et al. (2016). The study showed that the significant increase in DNA damage in iPSC motor neurons models was partially reversible by treating neurons with the antioxidant, Trolox. In three-month-old *C9orf72* patient iPSC-derived motor neurons from three patients (G4C2 > 590 repeats), treated with Trolox showed a partial reduction in DNA damage by comet assay (*p* < 0.01), compared with untreated neurons. Similarly, a partial reduction in DNA damage was also observed in iPSC-derived motor neurons transduced with poly-GR (80 repeats) following Trolox treatment (*p* < 0.01), compared with untreated neurons. These results suggest that targeting oxidative stress may represent a potential therapeutic approach for mitigating genome instability in C9orf72 models for ALS-FTD.

Another strategy was suggested by Walker et al. (2017), who evaluated whether targeting p62/SQSTM1, which can regulate the DDR through multiple mechanisms, or SETX that is required for R-loop resolution could alleviate DNA damage in a *C9orf72* DPR model. In MRC-5 fibroblasts expressing 69-V5-tagged poly-GA DPRs, depletion of p62/SQSTM1significantly reduced γH2AX foci (*p* = 0.0146). Similarly, *SETX* overexpression decreased γH2AX levels. Importantly, combing both approaches (*p62* KD and *SETX* OE) further reduced DSB. These results suggest that modulating p62/SQSTM1 expression or enhancing R-loop resolution are potential therapeutic strategies to mitigate the effects of accumulating DNA damage.

As described above, Lopez-Gonzalez et al. (2019) reported overactivation of Ku80/Ku70 dimer and downstream DDR signaling in *C9orf72* patient-derived neurons. Building on this, the authors demonstrated that a partial reduction in gene dosage of *Ku80* in two independent clones of *Ku80^+/−^* iPSC-derived MNs was sufficient to suppress the apoptotic cascade. Deletion of a single *Ku80* allele using CRISPR-Cas9 reduced *Ku80* expression by 50%. Comet assays performed with 2-month-old MNs revealed no increase in DNA damage – indeed one of the *Ku80*^+/−^ clones had DNA damage comparable to the parental *Ku80*^+/+^ line, whilst the second clone had a significant reduction in comet tail length (p < 0.01) and percentage of DNA in the tails (*p* < 0.05). This indicates that the residual Ku80/Ku70 complex in the clones was sufficient to repair DNA breaks but– importantly– the reduction in gene dosage significantly decreased activation of key DDR or apoptosis components, including pATM (*p* < 0.05), p-p53 (*p* < 0.05), PUMA (clone 1: *p* < 0.01, clone 2: *p* < 0.05), and cleaved caspase-3 (*p* < 0.05). Similarly, knockdown of *Ku80* using either lentiviral shRNA (50% KD) or self-delivering small interfering RNA (sdRNA) with lipophilic conjugates in the same patient iPSC-derived MNs model also significantly suppressed pro-apoptotic protein expression, including pATM (shRNA: *p* < 0.01), PUMA (shRNA *p* < 0.0001, sdRNA: *p* < 0.001), and cleaved caspase-3 (sdRNA: *p* < 0.001).

Maor-Nof et al. (2021) used two *C9orf72* models: mouse primary cortical neurons and *C9orf72* patient iPSC-derived MNs to demonstrate that p53 depletion is neuroprotective and reduces DNA damage. In embryonic primary cortical neurons transduced with lentiviruses expressing poly-PR (50 DPRs), *p53* KD markedly lower γH2AX levels compared to wildtype (*p* < 0.0001). Similarly, in *C9orf72* patient iPSC-derived MNs cultured for 2.5 months and their isogenic controls, *p53* KD *via* shRNA expression reduced DNA damage, as measured by comet assay with reduced comet tail length (*p* < 0.05) and percentage of DNA content in the tail (<40%, *p* < 0.05) relative to *C9orf72*-MNs expressing the control shRNA.

#### 3.6.6 Behavioral studies

Behavioral studies were conducted in two *C9orf72* mouse models to assess therapeutic efficacy (Maor-Nof et al., 2021; He et al., 2023) and in one study to examine neurodegeneration in mice injected with poly-GA (Walker et al., 2017). Targeting DDR and DNA repair factors was the most common approach, demonstrating positive effects on both lifespan and movement ability. Maor-Nof et al. (2021) injected *p53^+/−^* or *p53^−/−^* mouse intracerebroventricularly (ICV) with an AAV9 vector to express C9orf72 GFP-tagged-poly-PR (50 DPRs) or GFP only at post-natal day 0. The median lifespan for WT mice injected with AAV9-poly-PR was 39 days; heterozygosity for *p53* reduction rescued lifespan to a small extent (median lifespan 54 days), but the effect was more dramatic in *p53^−/−^* mice with a 2.5x longer lifespan, with some mice living up to 300 days. He et al. (2023) showed that intracerebroventricularly injection of AAV9-GFP-poly-GR (50 DPRs) into neonatal day 0 wild-type and *C9orf72^−/−^* mice led to progressive neurodegenerative phenotypes. Six months after injection, *C9orf72^−/−^* AAV-GFP-GR mice showed a significant reduction in body weight compared to *C9orf72^+/+^*AAV-GFP control mice (*p* < 0.05), along with impaired motor function, including decreased four-limb grip strength (*p* < 0.05) and poor performance on the accelerating rotarod test (*p* < 0.01). Histological analysis revealed a marked loss of NeuN-positive cortical neuron number (*p* < 0.001), reduced numbers of ChAT-positive motor neurons in the lumbar spinal cord (*p* < 0.01), and increased neuromuscular junction denervation in *C9orf72^−/−^* AAV-GFP-GR mice compared to wildtype mice injected with AAV9-poly-GR.

Walker et al. (2017) documented a neurodegenerative phenotype in mice injected with poly-GA DPRs. Compared to 0x poly-GA DPR controls, mice injected with 69x poly-GA DPRs displayed significant impairment at 6 months on the Catwalk system (*p* < 0.01), followed by progressive behavioral deficits at 12 months in the hind limb splay test (*p* < 0.01).

## 4 Discussion

DNA damage and impaired DDR are two mechanisms that are increasingly recognized as a hallmark for ALS and ALS-FTD. This systematic review investigated evidence across various models including cellular, animal, and patient-derived models. The included studies showed that *C9orf72* repeat expansions and DPRs impact genome integrity through multiple mechanisms, which in turn feed in disease progression.

### 4.1 Mechanisms of DNA damage accumulation

The systematic review found four major mechanisms that lead to increased DNA damage in *C9orf72* models. First, defective ATM signaling which was observed across models, with G4C2 and poly-GA DPRs suppressing both ATM activation and 53BP1 recruitment (Walker et al., 2017; Nihei et al., 2020). Mechanistically, this was due to sequestration of ATM and impaired RNF168-dependent ubiquitylation, resulting in diminished DDR signaling and abnormal nuclear localization of HDAC4 (Walker et al., 2017). These defects in the ATM-RNF168-53BP1 axis uncover a new neurodegenerative mechanism that extends previous findings of how ATM deficiency contributes to neurodegeneration (Lee and Paull, 2021; Paull and Woolley, 2024). Second, there was compromised DNA repair efficiency, mainly in the NHEJ and SSA pathways. Andrade et al. (2020) demonstrated that DPRs bound Nucleophosmin (NPM1), disrupting repair kinetics and increasing γH2AX levels. Consistent with this, He et al. (2023) reported an important function of *C9orf72* in stabilizing the DNA-PKcs-Ku70/Ku80 complex, supporting Lig4-XRCC4 assembly and loss of C9orf72 impairs this interaction, decreases Lig4 recruitment, and elevates DNA breaks, both *in vitro* and *in vivo* in *C9orf72*^−/−^ mice. Together, these results highlight a dual mechanism including gain-of-function toxicity from DPRs as well as loss of function of *C9orf72* in DNA repair. Third, accumulation of R-loops was documented in both *C9orf72* repeat expansion models and DPR models, with SETX overexpression reducing DNA breaks and toxicity (Walker et al., 2017). Although Pal et al. (2021) found no increase in R-loops in *C9orf72* patient iPSC-derived MNs despite the presence of DNA damage, this might be attributed to model-dependent variability and methods used to measure R-loop. Finally, mitochondrial dysfunction was reported as a driver of DNA damage. Lopez-Gonzalez et al. (2016) demonstrated that poly-GR, but not poly-GA expression, induced an increase in mitochondrial ROS and altered membrane potential, consistent with interactome data that showing preferential binding of GR to mitochondrial and ribosomal proteins. These DPR-specific effects help to unpick and understanding the role of oxidative stress in ALS models.

### 4.2 Downstream consequences: repeat expansion

Kojak et al. (2024) reported that DNA strand breaks themselves accelerated C9orf72 repeat expansion via Rad51-mediated homologous recombination and Msh2-driven mismatch repair. This suggests that DNA damage initiates a feed-forward loop that amplifies repeat instability. In parallel, activation of the STING pathway was detected in vulnerable neurons in both patient and mouse models (Marques et al., 2024). The activation of STING pathway was strongly detected in Betz cells and spinal MNs, with activation of both STING pathways; canonical (IRF3) and non-canonical (NF- κB). These findings may contribute to the existing body of evidence regarding the dysregulation of immunity in ALS disease, highlighting nuclear DNA damage as a key factor that trigger innate immune responses (Tan et al., 2022; Huang et al., 2023).

Our review contributes to the field in three ways. First, it shows DNA damage in ALS-FTD stems from a combination of ATM inhibition, NHEJ impairment, R-loop toxicity, and oxidative stress. Second, it helps to understand the *C9orf72* dilemma by demonstrating that C9orf72 loss of function affects repair complexes, while the DPRs impact on key DDR factors. Third, it reveals that DNA damage is a primary driver of *C9orf72* G4C2 repeat expansion as well as immune activation. On the other hand, the body of evidence remains heterogeneous many studies mainly used non-neuronal cell lines. While these models provide mechanistic insights, they still do not capture neuronal vulnerability, if neurons are especially vulnerable. iPSC-derived neurons exhibited differences in differentiation and maturity, which could lead to inconsistency in results. Finally, the sensitivity of methods used to detect DNA damage and R-loops varied.

### 4.3 C9orf72 loss of function, repeat expansions, and DPR-induced DNA damage

Two suggested mechanisms that link to *C9orf72* pathology impact genome integrity: loss- and gain-of-function mechanisms. Loss-of-function of *C9orf72* impacts on DSB repair by destabilizing DNA-PKcs-Ku70/Ku80 complexes, reducing levels of pDNA-PKcs and γH2AX despite the significant increase in DNA breaks. This places the C9orf72 protein as a direct scaffold in the DDR, in addition to its known functions in trafficking and autophagy (Pal et al., 2021; He et al., 2023). On the other hand, *C9orf72* gain-of-function mechanisms, which includes both repeat expansions and DPRs further elevate genome instability in neurons. In iPSC-derived MNs from patients, the accumulation of DNA damage as measured by with γH2AX and DNA repair factors occurred with increasing neuronal age but axonal transport defects were apparent earlier, indicating that DNA damage may amplify rather than initiate neuronal dysfunction (Pal et al., 2021). RNA foci colocalize with γH2AX in patient neurons, and DPRs, including poly-GA, poly-GR, and poly-PR consistently elevated DNA DSBs while interfering with ATM activation and 53BP1 recruitment to damage sites (Walker et al., 2017; Farg et al., 2017; Nihei et al., 2020; Chang et al., 2024). Moreover, hnRNPA3 depletion further accelerate DPR toxicity, which may link RNA-binding protein dysfunction to impaired DNA repair (Nihei et al., 2020).

### 4.4 Therapeutic modulation of DNA damage

Therapeutic interventions support a causal role of DNA damage in *C9orf72* pathology. Three suggested therapeutic mechanisms were found in the included studies: the antioxidant Trolox reduced DNA breaks (Lopez-Gonzalez et al., 2016); *SETX* overexpression reduced R-loop induced lesions (Walker et al., 2017); and depletion of either Ku80 or p53 suppressed pro-apoptotic pathways and improved neuronal survival (Lopez-Gonzalez et al., 2019; Maor-Nof et al., 2021). These findings indicated that targeting the DDR or DNA repair pathways may enhance lifespan and locomotion and this approach has potential as a therapeutic strategy.

## 5 Conclusion

This is the first systematic review to our knowledge that looked specifically at the contribution of DNA damage to *C9orf72*-based ALS-FTD pathology. This review provides a foundation for understanding disease progression, future clinical applications, and potential therapeutic strategies. A meta-analysis of included studies was prevented by considerable differences in study design and the way that outcomes were reported, most notably for experiments using *in vivo* animal models: there is a strong argument for standardizing methodology to allow comparisons. Several limitations must be considered when interpreting the results. Firstly, the predominant emphasis of studies was on *C9orf72* DPRs and with only limited investigations of the alternative proposed mechanisms of *C9orf72* related loss-of-function and RREs. Secondly, there were a limited number of studies available for certain DPRs with no study that investigated the role of poly-PA in DNA damage, which makes quantitative measurements of DNA damage challenging. Finally, as previously discussed in the accompanying systematic reviews of TDP43-related and FUS-related ALS-FTD (Almalki et al., 2025b; Almalki et al., 2025c), there is a need for the standardization of methods for assessing DNA damage.

## Data Availability

The original contributions presented in the study are included in the article/Supplementary material, further inquiries can be directed to the corresponding author.

## References

[ref1] Almalki S Salama M Taylor MJ Ahmed Z. (2025a). C9orf72-related amyotrophic lateral sclerosis-frontotemporal dementia and links to the DNA damage response: a systematic review. Front. Mol. Neurosci. 18:1671906. doi: 10.3389/fnmol.2025.1671906 PMC12615431 41245603

[ref2] Almalki S Salama M Taylor M. J. Ahmed Z Tuxworth I. (2025b). TDP-43 related amyotrophic lateral sclerosis-frontotemporal dementia and links to the DNA damage response: a systematic review. Front. Mol. Neurosci. 18:1671909. doi: 10.3389/fnmol.2025.1671909 PMC12615431 41245603

[ref3] Almalki S Salama M Taylor MJ Ahmed Z Tuxworth I. F. (2025c). FUS-related amyotrophic lateral sclerosis-frontotemporal dementia and links to the DNA damage response: a systematic review. Front. Mol. Neurosci. 18:1671910. doi: 10.3389/fnmol.2025.1671910 41245603 PMC12615431

[ref4] Andrade N. S. Ramic M. Esanov R. Liu W. Rybin M. J. Gaidosh G. . (2020). Dipeptide repeat proteins inhibit homology-directed DNA double strand break repair in C9ORF72 ALS/FTD. Mol. Neurodegener. 15:13. doi: 10.1186/s13024-020-00365-9, PMID: 32093728 PMC7041170

[ref5] Balendra R. Isaacs A. M. (2018). C9orf72-mediated ALS and FTD: multiple pathways to disease. Nat. Rev. Neurol. 14, 544–558. doi: 10.1038/s41582-018-0047-2, PMID: 30120348 PMC6417666

[ref6] Chang Y. J. Lin K. T. Shih O. Yang C. H. Chuang C. Y. Fang M. H. . (2024). Sulfated disaccharide protects membrane and DNA damages from arginine-rich dipeptide repeats in ALS. Sci. Adv. 10:eadj0347. 38394210 10.1126/sciadv.adj0347 PMC10889363

[ref7] Chatterjee N. Walker G. C. (2017). Mechanisms of DNA damage, repair, and mutagenesis. Environ. Mol. Mutagen. 58, 235–263. 28485537 10.1002/em.22087 PMC5474181

[ref8] Cohen S. Puget N. Lin Y. L. Clouaire T. Aguirrebengoa M. Rocher V. . (2018). Senataxin resolves RNA:DNA hybrids forming at DNA double-strand breaks to prevent translocations. Nat. Commun. 9:533. doi: 10.1038/s41467-018-02894-w, PMID: 29416069 PMC5803260

[ref9] De Waard M. C. Van Der Pluijm I. Zuiderveen Borgesius N. Comley L. H. Haasdijk E. D. Rijksen Y. . (2010). Age-related motor neuron degeneration in DNA repair-deficient Ercc1 mice. Acta Neuropathol. 120, 461–475. doi: 10.1007/s00401-010-0715-9, PMID: 20602234 PMC2923326

[ref10] Deng Q. Holler C. J. Taylor G. Hudson K. F. Watkins W. Gearing M. . (2014). FUS is phosphorylated by DNA-PK and accumulates in the cytoplasm after DNA damage. J. Neurosci. 34, 7802–7813. doi: 10.1523/JNEUROSCI.0172-14.2014, PMID: 24899704 PMC4044245

[ref11] Fang M. Deibler S. K. Nana A. L. Vatsavayai S. C. Banday S. Zhou Y. . (2023). Loss of TDP-43 function contributes to genomic instability in amyotrophic lateral sclerosis. Front. Neurosci. 17:17. doi: 10.3389/fnins.2023.1251228, PMID: 37849894 PMC10577185

[ref12] Farg M. A. Konopka A. Soo K. Y. Ito D. Atkin J. D. (2017). The DNA damage response (DDR) is induced by the C9orf72 repeat expansion in amyotrophic lateral sclerosis. Hum. Mol. Genet. 26, 2882–2896. doi: 10.1093/hmg/ddx170, PMID: 28481984

[ref13] Gianini M. Bayona-Feliu A. Sproviero D. Barroso S. I. Cereda C. Aguilera A. (2020). TDP-43 mutations link amyotrophic lateral sclerosis with R-loop homeostasis and R loopmediated DNA damage. PLoS Genet. 16:e1009260. doi: 10.1371/journal.pgen.1009260 33301444 PMC7755276

[ref14] Gong J. Huang M. Wang F. Ma X. Liu H. Tu Y. . (2017). RBM45 competes with HDAC1 for binding to FUS in response to DNA damage. Nucleic Acids Res. 45, 12862–12876. doi: 10.1093/nar/gkx1102, PMID: 29140459 PMC5728411

[ref15] Guerrero E. N. Mitra J. Wang H. Rangaswamy S. Hegde P. M. Basu P. . (2019). Amyotrophic lateral sclerosis-associated TDP-43 mutation Q331K prevents nuclear translocation of XRCC4-DNA ligase 4 complex and is linked to genome damage-mediated neuronal apoptosis. Hum. Mol. Genet. 28, 2459–2476. doi: 10.1093/hmg/ddz062, PMID: 31067307 PMC6659010

[ref17] He L. Liang J. Chen C. Chen J. Shen Y. Sun S. . (2023). C9orf72 functions in the nucleus to regulate DNA damage repair. Cell Death Differ. 30, 716–730. doi: 10.1038/s41418-022-01074-0, PMID: 36220889 PMC9984389

[ref18] Hooijmans C. R. Rovers M. M. De Vries R. B. M. Leenaars M. Ritskes-Hoitinga M. Langendam M. W. (2014). SYRCLE’S risk of bias tool for animal studies. BMC Med. Res. Methodol. 14, 43–52. doi: 10.1186/1471-2288-14-43, PMID: 24667063 PMC4230647

[ref19] Huang Y. Liu B. Sinha S. C. Amin S. Gan L. (2023). Mechanism and therapeutic potential of targeting cGAS-STING signaling in neurological disorders. Mol. Neurodegener. 18:79. doi: 10.1186/s13024-023-00672-x, PMID: 37941028 PMC10634099

[ref20] Jia W. Kim S. H. Scalf M. A. Tonzi P. Millikin R. J. Guns W. M. . (2021). Fused in sarcoma regulates DNA replication timing and kinetics. J. Biol. Chem. 297:101049. doi: 10.1016/j.jbc.2021.101049, PMID: 34375640 PMC8403768

[ref21] Junghans M. John F. Cihankaya H. Schliebs D. Winklhofer K. F. Bader V. . (2022). ROS scavengers decrease γH2ax spots in motor neuronal nuclei of ALS model mice in vitro. Front. Cell. Neurosci. 16:963169. doi: 10.3389/fncel.2022.963169, PMID: 36119129 PMC9470831

[ref22] Karanjawala Z. E. Hsieh C. L. Lieber M. R. (2003). Overexpression of cu/Zn superoxide dismutase is lethal for mice lacking double-strand break repair. DNA Repair 2, 285–294. doi: 10.1016/S1568-7864(02)00218-5, PMID: 12547391

[ref23] Kim B. W. Jeong Y. E. Wong M. Martin L. J. (2020). DNA damage accumulates and responses are engaged in human ALS brain and spinal motor neurons and DNA repair is activatable in iPSC-derived motor neurons with SOD1 mutations. Acta Neuropathol. Commun. 8:7. doi: 10.1186/s40478-019-0874-4, PMID: 32005289 PMC6995159

[ref24] Kodavati M. Wang H. Guo W. Mitra J. Hegde P. M. Provasek V. . (2024). FUS unveiled in mitochondrial DNA repair and targeted ligase-1 expression rescues repair-defects in FUS-linked motor neuron disease. Nat. Commun. 15:2156. doi: 10.1038/s41467-024-45978-6, PMID: 38461154 PMC10925063

[ref25] Kojak N. Kuno J. Fittipaldi K. E. Khan A. Wenger D. Glasser M. . (2024). Somatic and intergenerational G4C2 hexanucleotide repeat instability in a human C9orf72 knock-in mouse model. Nucleic Acids Res. 52, 5732–5755. doi: 10.1093/nar/gkae250, PMID: 38597682 PMC11162798

[ref26] Konopka A. Whelan D. R. Jamali M. S. Perri E. Shahheydari H. Toth R. P. . (2020). Impaired NHEJ repair in amyotrophic lateral sclerosis is associated with TDP-43 mutations. Mol. Neurodegener. 15:51. doi: 10.1186/s13024-020-00386-4, PMID: 32907630 PMC7488163

[ref27] Lee J. H. Paull T. T. (2021). Cellular functions of the protein kinase ATM and their relevance to human disease. Nat. Rev. Mol. Cell Biol.. Nature Research 22, 796–814. 34429537 10.1038/s41580-021-00394-2

[ref28] Lee Y. J. Rio D. C. (2024). A mutation in the low-complexity domain of splicing factor hnRNPA1 linked to amyotrophic lateral sclerosis disrupts distinct neuronal RNA splicing networks. Genes Dev. 38, 11–30. doi: 10.1101/gad.351104.123, PMID: 38182429 PMC10903937

[ref29] Levone B. R. Lenzken S. C. Antonaci M. Maiser A. Rapp A. Conte F. . (2021). FUS-dependent liquid-liquid phase separation is important for DNA repair initiation. J. Cell Biol. 220:e202008030. doi: 10.1083/jcb.202008030, PMID: 33704371 PMC7953258

[ref30] Lopez-Gonzalez R. Lu Y. Gendron T. F. Karydas A. Tran H. Yang D. . (2016). Poly(GR) in C9ORF72-related ALS/FTD compromises mitochondrial function and increases oxidative stress and DNA damage in iPSC-derived motor neurons. Neuron 92, 383–391. doi: 10.1016/j.neuron.2016.09.015, PMID: 27720481 PMC5111366

[ref31] Lopez-Gonzalez R. Yang D. Pribadi M. Kim T. S. Krishnan G. Choi S. Y. . (2019). Partial inhibition of the overactivated Ku80-dependent DNA repair pathway rescues neurodegeneration in C9ORF72-ALS/FTD. Proc. Natl. Acad. Sci. USA 116, 9628–9633. doi: 10.1073/pnas.1901313116, PMID: 31019093 PMC6511021

[ref32] Maor-Nof M. Shipony Z. Lopez-Gonzalez R. Nakayama L. Zhang Y. J. Couthouis J. . (2021). p53 is a central regulator driving neurodegeneration caused by C9orf72 poly(PR). Cell 184, 689–708.e20. doi: 10.1016/j.cell.2020.12.025, PMID: 33482083 PMC7886018

[ref33] Marques C. Held A. Dorfman K. Sung J. Song C. Kavuturu A. S. . (2024). Neuronal STING activation in amyotrophic lateral sclerosis and frontotemporal dementia. Acta Neuropathol. 147:56. doi: 10.1007/s00401-024-02688-z, PMID: 38478117 PMC10937762

[ref34] Martin L. J. (2008). DNA damage and repair: relevance to mechanisms of neurodegeneration. J. Neuropathol. Exp. Neurol. 67, 377–387. doi: 10.1097/NEN.0b013e31816ff780, PMID: 18431258 PMC2474726

[ref35] Martin L. J. Liu Z. Chen K. Price A. C. Yan P. Swaby J. A. . (2007). Motor neuron degeneration in amyotrophic lateral sclerosis mutant superoxide dismutase-1 transgenic mice: mechanisms of mitochondriopathy and cell death. J. Comp. Neurol. 500, 20–46. 17099894 10.1002/cne.21160

[ref36] Mithal N. P. Radunovic A. Figlewicz D. A. Mcmillan T. J. Leigh P. N. (1999). Cells from individuals with SOD-1 associated familial amyotrophic lateral sclerosis do not have an increased susceptibility to radiation-induced free radical production or DNA damage. J. Neurol. Sci. 164, 82–92. 10385054 10.1016/s0022-510x(99)00053-2

[ref37] Mitra J. Guerrero E. N. Hegde P. M. Liachko N. F. Wang H. Vasquez V. . (2019). Motor neuron disease-associated loss of nuclear TDP-43 is linked to DNA double-strand break repair defects. Proc. Natl. Acad. Sci. USA 116, 4696–4705. doi: 10.1073/pnas.1818415116, PMID: 30770445 PMC6410842

[ref38] Mitra J. Kodavati M. Dharmalingam P. Guerrero E. N. Rao K. S. Garruto R. M. . (2025). Endogenous TDP-43 mislocalization in a novel knock-in mouse model reveals DNA repair impairment, inflammation, and neuronal senescence. Acta Neuropathol. Commun. 13:54, PMID: 40057796 10.1186/s40478-025-01962-9 PMC11889789

[ref39] Naumann M. Pal A. Goswami A. Lojewski X. Japtok J. Vehlow A. . (2018). Impaired DNA damage response signaling by FUS-NLS mutations leads to neurodegeneration and FUS aggregate formation. Nat. Commun. 9:335. doi: 10.1038/s41467-017-02299-1, PMID: 29362359 PMC5780468

[ref40] Nihei Y. Mori K. Werner G. Arzberger T. Zhou Q. Khosravi B. . (2020). Poly-glycine–alanine exacerbates C9orf72 repeat expansion-mediated DNA damage via sequestration of phosphorylated ATM and loss of nuclear hnRNPA3. Acta Neuropathol. 139, 99–118. doi: 10.1007/s00401-019-02082-0, PMID: 31642962 PMC6942035

[ref41] Niu Y. Pal A. Szewczyk B. Japtok J. Naumann M. Glaß H. . (2024). Cell-type-dependent recruitment dynamics of FUS protein at laser-induced DNA damage sites. Int. J. Mol. Sci. 25:3526. doi: 10.3390/ijms25063526, PMID: 38542501 PMC10971167

[ref42] Nogami M. Sano O. Adachi-Tominari K. Hayakawa-Yano Y. Furukawa T. Iwata H. . (2022). DNA damage stress-induced translocation of mutant FUS proteins into cytosolic granules and screening for translocation inhibitors. Front. Mol. Neurosci. 15:15. doi: 10.3389/fnmol.2022.953365, PMID: 36606141 PMC9808394

[ref16] Office of Health Assessment and Translation (OHAT) Division of the National Toxicology Program National Institute of Environmental Health Sciences. (2019). Handbook for conducting a literature-based health assessment using OHAT approach for systematic review and evidence integration.

[ref7001] Page M. J. McKenzie J. E. Bossuyt P. M. Boutron I. Hoffmann T. C. Mulrow C. D. . (2021). The PRISMA 2020 statement: an updated guideline for reporting systematic reviews. BMJ. 372:n71. doi: 10.1136/bmj.n71 33782057 PMC8005924

[ref43] Pal A. Kretner B. Abo-Rady M. Glab H. Dash B. P. Naumann M. . (2021). Concomitant gain and loss of function pathomechanisms in C9ORF72 amyotrophic lateral sclerosis. Life Sci. Alliance. 4:e202000764. doi: 10.26508/lsa.202000764 33619157 PMC7918691

[ref44] Paull T. T. Woolley P. R. (2024). A-T neurodegeneration and DNA damage-induced transcriptional stress. DNA Repair (Amst) 135:103647. doi: 10.1016/j.dnarep.2024.103647, PMID: 38377644 PMC11707827

[ref45] Penndorf D. Tadić V. Witte O. W. Grosskreutz J. Kretz A. (2017). DNA strand breaks and TDP-43 mislocation are absent in the murine hSOD1G93A model of amyotrophic lateral sclerosis in vivo and in vitro. PLoS One 12:e0183684. doi: 10.1371/journal.pone.0183684, PMID: 28832631 PMC5568271

[ref46] Provasek V. E. Bacolla A. Rangaswamy S. Mitra J. Kodavati M. Yusuf I. O. RNA/DNA binding protein TDP43 regulates DNA mismatch repair genes with implications for genome stability. (2024) 10.1093/nar/gkaf920 PMC12455596 40985771

[ref47] Richard P. Feng S. Tsai Y. L. Li W. Rinchetti P. Muhith U. . (2020). SETX (senataxin), the helicase mutated in AOA2 and ALS4, functions in autophagy regulation. Autophagy, 17, 1889–1906. doi: 10.1080/15548627.2020.1796292 32686621 PMC8386630

[ref48] Tamaki Y. Ross J. P. Alipour P. Castonguay C. É. Li B. Catoire H. . (2023). Spinal cord extracts of amyotrophic lateral sclerosis spread TDP-43 pathology in cerebral organoids. PLoS Genet. 19:e1010606. doi: 10.1371/journal.pgen.1010606, PMID: 36745687 PMC9934440

[ref49] Tan H. Y. Yong Y. K. Xue Y. C. Liu H. Furihata T. Shankar E. M. . (2022). cGAS and DDX41-STING mediated intrinsic immunity spreads intercellularly to promote neuroinflammation in SOD1 ALS model. IScience 25:104404. doi: 10.1016/j.isci.2022.104404, PMID: 35712074 PMC9194172

[ref50] Tang X. Toro A. Sahana T. G. Gao J. Chalk J. Oskarsson B. E. . (2020). Divergence, convergence, and therapeutic implications: a cell biology perspective of C9ORF72-ALS/FTD. Mol. Neurodegener. 15:34. doi: 10.1186/s13024-020-00383-7 32513219 PMC7282082

[ref51] Tzeplaeff L. Wilfling S. Requardt M. V. Herdick M. (2023). Current state and future directions in the therapy of ALS. Cells 12:1523. doi: 10.3390/cells12111523, PMID: 37296644 PMC10252394

[ref52] Walker C. Herranz-Martin S. Karyka E. Liao C. Lewis K. Elsayed W. . (2017). C9orf72 expansion disrupts ATM-mediated chromosomal break repair. Nat. Neurosci. 20, 1225–1235. doi: 10.1038/nn.4604, PMID: 28714954 PMC5578434

[ref53] Wang W. Y. Pan L. Su S. C. Quinn E. J. Sasaki M. Jimenez J. C. . (2013). Interaction of FUS and HDAC1 regulates DNA damage response and repair in neurons. Nat. Neurosci. 16, 1383–1391. doi: 10.1038/nn.3514, PMID: 24036913 PMC5564396

[ref54] Wang H. Rangaswamy S. Kodavati M. Mitra J. Guo W. Guerrero E. N. . (2019). RT2 PCR array screening reveals distinct perturbations in DNA damage response signaling in FUS-associated motor neuron disease. Mol. Brain 12:103. doi: 10.1186/s13041-019-0526-4, PMID: 31801573 PMC6894127

