# Supplementary material for: C9orf72-related amyotrophic lateral sclerosis-frontotemporal dementia and links to the DNA damage response: a systematic review
Source: Front Mol Neurosci. 2025 Nov 18;18:1671906. doi: 10.3389/fnmol.2025.1671906 (PMC12669223; doi:10.3389/fnmol.2025.1671906)
Supplement: Supplementary file 1 [file Table_1.docx]

Supplementary Material

# Supplementary Data

This supplementary analysis accompanies our systematic review that examined the accumulation of DNA damage and disruptions in the DNA damage response (DDR) and DNA repair pathways across models of ALS-FTD. The main review identified 41 relevant publications with the majority focused on C9orf72, TDP-43, and FUS; these were analyzed separately in three manuscripts: Almalki *et al.* 2025a, b and c (1–3). Eleven studies investigated five additional ALS-FTD-associated genes: *EWS, TAF15*, *SOD1*, *SETX*, and *hnRNPA1* plus the Wobbler mouse models of ALS which carries a mutation in *Vps54*. EWS and TAF15 are members of the FET family of proteins along with FUS and hence, were included with FUS in the main review (Almalki *et al.* 2025c (3)). Data on *SOD1*, *SETX*, *hnRNPA1*, and *Vps54* (nine studies) (4–12) are presented here in this supplementary section.

**1. *SOD1***

*SOD1* encodes superoxide dismutase 1, an enzyme that protects cells from oxidative stress by catalyzing the conversion of superoxide radicals into oxygen and hydrogen peroxide. Mutations in SOD1 are linked to familial ALS, contributing to toxic protein aggregation and mitochondrial dysfunction (13) while its role in FTD remains less well-established. A total of five studies, with two conducted in Europe (6,8) and three in North America (4,7,9), explored the involvement of DNA damage, the DNA damage response (DDR), and DNA repair mechanisms in the context of *SOD1*  proteinopathy across both neuronal and non-neuronal models. Three experimental models employed in this study: cell lines, iPSC derived neurons, and transgenic mouse models (Table 1). Several *SOD1* mutations were examined, with the G93A mutation being the primary focus in three studies (4,7,8). The A4V mutation was investigated in two studies (4,6), while the Gly37Arg, and Ile149Thr mutations (6) and *SOD1* overexpression (OE) (9) were each investigated in one study.

γH2AX staining was the primary assay utilized to evaluate the accumulation of DNA damage (4,8). Several other assays, including TUNEL, PFGE, and the comet assay, were also employed, albeit less frequently.

**1.1 Primary outcome: Detection or accumulation of DNA damage in the CNS**

Three studies (4,6,8) demonstrated no significant increase in DNA damage in several fALS SOD1 mutations compared to control groups. Kim *et.al.* 2020 (4) used patient derived SOD1-A4V iPSC and healthy iPSC edited via CRISPR-Cas9 to carry the G39A mutation. Both cells differentiated into spinal motor neurons. Mithal *et.al.* 1999 (6) analyzed lymphoblastoid cell lines harboring one of three fALS SOD1 mutations (Ala4Val/ Gly37Arg/Ile149Thr ). Penndorf *et.al.* 2017 (8) assessed spinal cord tissue from transgenic mice overexpressing the hSOD1 G93A mutation. Although DNA damage was induced in these models using genotoxic agents, no further increase above that detected in the controls. Furthermore, there was no significant difference in DNA damage observed between astrocytes and motor neurons derived from SOD1 G39A transgenic mice. In contrast, Martin *et al.* 2007 (7) reported formation of single strand breaks (SSBs) as a precursor to double strand breaks (DSBs) occurring in G93A-mSOD1 transgenic mice at 9 weeks, which corresponds to the early symptomatic stages, and affecting both mitochondrial and nuclear DNA.

**1.2 Secondary outcome:** **DNA repair pathways**

One study (9) investigated the impact of overexpression of *SOD1* in Ku86-deficient mice. Karanjawala *et al.* 2003 (9) reported that overexpression of human wild type *SOD1* in Ku86⁺/⁺ mice or in heterozygous Ku86⁺/⁻ mice in which the non-homologous end joining (NHEJ) repair pathway is partially dysfunctional did not increase neuronal cell death compared to homozygous mutant Ku86⁻/⁻ mice with are deficient for NHEJ activity (p = 0.01). This indicates that *SOD1* overexpression alone is not toxic in these models. Interestingly, some Ku86⁻/⁻ mice with overexpression of wild type human *SOD1* survived despite similar levels of SOD1 protein due to the presence of protective alleles on mouse chromosome 13.

**2. *SETX***

*SETX* encodes Senataxin, a DNA/RNA helicase involved in resolving R-loops and facilitating transcription termination and DNA repair. *SETX* mutations are linked to ALS4, a rare juvenile-onset form of ALS (10,11). Similar to *SOD1,* *SETX* association to FTD is not well defined. Two studies focused on *SETX*, conducted in USA and France (10,11). Both used immortalised cell lines as a model of *SETX* deficiency (U87 and U2OS cells, respectively) (Table 2). The two studies used immunofluorescence to assess levels of DNA damage as well as ChIP-seq and DRIP-seq.

**2.1 Primary outcome: Detection or accumulation of DNA damage in the CNS**

The two studies reported an accumulation of DNA damage in cell lines using γH2AX assay as a marker of double-strand breaks (DSBs). Richard *et al.* 2020 (10) reported a significant increase in γH2AX protein levels following *SETX* knockdown combined with nutrient starvation to induce autophagy in U87 glioblastoma astrocytoma cells, showing that accumulation of DNA damage associated with autophagy impairment. Similarly, Cohen *et al.* 2018 (11) demonstrated that *SETX* depletion led to a 7% increase in R-loop formation around DSB sites, as well as an elevation in DDR factors including γH2AX and 53BP1. Furthermore, induction of DNA damage through etoposide or irradiation further exacerbated the damage.

**2.2 Secondary outcome:** **DNA repair pathways**

The impact of *SETX* KD on DNA repair mechanism was documented in one study by Cohen *et al.* 2018 (11). U2OS cells exhibited a reduction in the recruitment of RAD51 to DSBs and a mild reduction in the single strand annealing (SSA) repair pathway.

**3. hnRNPA1**

*hnRNPA1* encodes heterogeneous nuclear Ribonucleoprotein A1 (hnRNPA1), an RNA-binding protein involved in pre-mRNA splicing and RNA transport. Mutations in hnRNPA1 have been linked to ALS, while its role in FTD remains less well characterized (12).

One USA-based study investigated the role of *hnRNPA1* (12) using two biological models: FLP-In-293 cells and SH-SY5Y neuroblastoma cells (Table 3). One mutation linked to hnRNPA1, D262V, was investigated. RNA-sequencing test was conducted on both models to study alteration in gene splicing between hnRNPA1 D262V v*s*. wild-type. Additionally, TMT mass spectrometry was used to study changes in protein-protein interactions between the control and mutant cells for proteins involved in DNA damage response.

**3.1 Primary outcome: Detection or accumulation of DNA damage in the CNS**

RNA-seq analysis of FLP-In-293 cells showed that the *hnRNPA1* D262V mutation led to 2,240 splicing events affecting 1,532 genes, while in SH-SY5Y cells, 4,368 events impacted 3,322 genes. GO term enrichment analysis indicated these splicing changes were mainly related to genes involved in DDR, DNA repair, and chromatin remodeling processes.

**3.2 Secondary outcome:** **DNA repair pathways**

Spectrometry results demonstrated that the interaction between hnRNPA1 D262V protein and the DDR was altered. Further examination using irCLIP revealed that this mutation influenced RNA binding patterns, which corresponded with alterations in splicing of genes associated with DNA repair.

**4. *Vps54***

*Vps54* encode a vacuolar ATPase localized primarily to the Golgi. A point mutation in *Vps54* occurs in the Wobbler mouse that undergoes ALS-like neurodegeneration. One study carried out in Germany examined DNA damage accumulation in the Wobbler mouse (Table 4).

**4.1 Primary outcome: Detection or accumulation of DNA damage in the CNS**

Junghans *et al.* 2022 (5) reported increased DNA damage and DDR proteins in cervical spinal cord tissue of Wobbler mouse, with both DDR factors (p53BP1 and γH2AX ) significantly elevated at mRNA and protein levels (p < 0.01). Isolated primary motor neurons also showed accumulating DSB and increased ROS.

**Supplementary Discussion**

Accumulation of DNA damage was a near universal finding in the included studies focusing on C9orf72, TDP-43 and FUS. However, this was not the case for SOD1 where three of the five studies found no significant difference in DNA damage levels (4,6,8). Impairment of the DDR and DNA repair pathways were investigated in only one study (7), but taken together suggest that the fundamental disease mechanisms may be different for SOD1-related ALS-FTD. Most of studies focusing on *SOD1* relied exclusively on cell culture models. The one *in vivo* rodent study (9), which investigated the impact of *SOD1* overexpression in Ku86-deficient mice did not examine the effects of *SOD1* depletion or disease-relevant mutations on animal behaviour.

All nine studies included in this supplementary analysis were conducted primarily *in vitro* and were generally rated as having low risk of bias (RoB) reported by the OHAT tool. The sole *in vivo* study on *SOD1* (9) was assessed using the SYRCLE tool and showed high risk in seven domains, including blinding of the outcome, randomisation, allocation concealment, and outcome assessment, and the conclusions should be viewed in light of this potential risk.

**Supplementary Conclusion**

This is the first systematic review to our knowledge to investigate the involvement of DNA damage in ALS-FTD-associated genes, including the less-studied genes such as *TAF15* and *EWS*. The findings reported here together with the conclusions of the accompanying three main reports, demonstrate a strong association between DNA damage accumulation and genes implicated in ALS-FTD pathogenesis for the majority of genes studied, including these less well-understood genes, and indicate that DNA damage accumulation in the CNS may be considered a hallmark of ALS-FTD. The possible exception is *SOD1-*related ALS-FTD, where DNA damage may be of lesser importance.

However, several limitations should be considered when interpreting these results. The number of studies available for each gene was low, with some genes represented by only one or two publications. Additionally, there was a limited range of mutations investigated with a lack of model diversity when compared to studies focusing on TDP-43, C9orf72, and FUS, for which five distinct models, including ALS-specific iPSC cells and animal models, were used to permit more general conclusions to be reached. Finally, there is a critical need for greater assay diversity to assess DNA damage, standardized protocols for DNA damage evaluation, temporal analyses, and improved quantitative reporting.

**Supplementary References**

1. Almalki S, Salama M, Taylor MJ, Ahmed Z. C9orf72-related amyotrophic lateral sclerosis-frontotemporal dementia and links to the DNA Damage Response: a systematic review. 2025.

2. Almalki S, Salama M, Taylor MJ, Ahmed Z, Tuxworth I. TDP-43 related amyotrophic lateral sclerosis-frontotemporal dementia and links to the DNA Damage Response: a systematic review. 2025.

3. Almalki S, Salama M, Taylor MJ, Ahmed Z, Tuxworth I. FUS-related amyotrophic lateral sclerosis-frontotemporal dementia and links to the DNA Damage Response: a systematic review. 2025.

4. Kim BW, Jeong YE, Wong M, Martin LJ. DNA damage accumulates and responses are engaged in human ALS brain and spinal motor neurons and DNA repair is activatable in iPSC-derived motor neurons with SOD1 mutations. Acta Neuropathol Commun. 2020 Jan 31;8(1).

5. Junghans M, John F, Cihankaya H, Schliebs D, Winklhofer KF, Bader V, et al. ROS scavengers decrease γH2ax spots in motor neuronal nuclei of ALS model mice in vitro. Front Cell Neurosci. 2022 Aug 31;16.

6. Mithal NP, Radunovic A, Figlewicz DA, Mcmillan TJ, Leigh PN. Cells from individuals with SOD-1 associated familial amyotrophic lateral sclerosis do not have an increased susceptibility to radiation-induced free radical production or DNA damage. Vol. 164, Journal of the Neurological Sciences. 1999.

7. Martin LJ, Liu Z, Chen K, Price AC, Yan P, Swaby JA, et al. Motor neuron degeneration in amyotrophic lateral sclerosis mutant superoxide dismutase-1 transgenic mice: Mechanisms of mitochondriopathy and cell death. Journal of Comparative Neurology. 2007 Jan 1;500(1):20–46.

8. Penndorf D, Tadić V, Witte OW, Grosskreutz J, Kretz A. DNA strand breaks and TDP-43 mislocation are absent in the murine hSOD1G93A model of amyotrophic lateral sclerosis in vivo and in vitro. PLoS One. 2017 Aug 1;12(8).

9. Karanjawala ZE, Hsieh CL, Lieber MR. Overexpression of Cu/Zn superoxide dismutase is lethal for mice lacking double-strand break repair. Vol. 2, DNA Repair. 2003.

10. Richard P, Feng S, Tsai YL, Li W, Rinchetti P, Muhith U, et al. SETX (senataxin), the helicase mutated in AOA2 and ALS4, functions in autophagy regulation. Autophagy. 2020;1–18.

11. Cohen S, Puget N, Lin YL, Clouaire T, Aguirrebengoa M, Rocher V, et al. Senataxin resolves RNA:DNA hybrids forming at DNA double-strand breaks to prevent translocations. Nat Commun. 2018 Dec 1;9(1).

12. Lee YJ, Rio DC. A mutation in the low-complexity domain of splicing factor hnRNPA1 linked to amyotrophic lateral sclerosis disrupts distinct neuronal RNA splicing networks. Genes Dev. 2024 Feb 13;38(1–2):11–30.

13. Rosen DR, Siddiquet T, Pattersont D, Figlewicz DA, Sapp P, Hentatit A, et al. Mutations in Cu/Zn superoxide dismutase gene are associated with familial amyotrophic lateral sclerosis. 1993.

# Supplementary Figures and Tables

## Supplementary Figures

Supplementary Figure 1. PRISMA flow chart for the genes included in the supplementary analysis (*SOD1, SETX, hnRNPA1,* and *Vps54*).


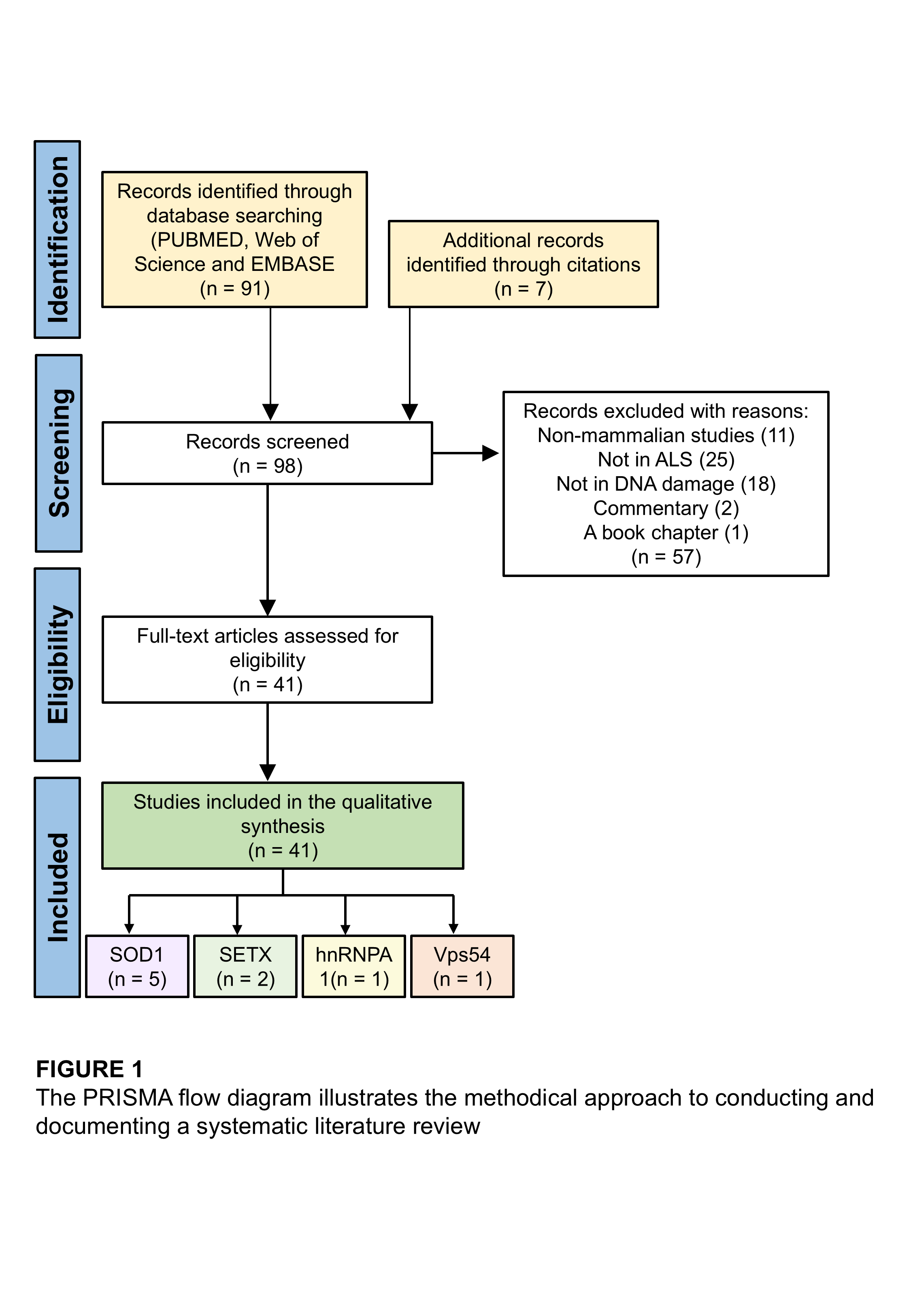


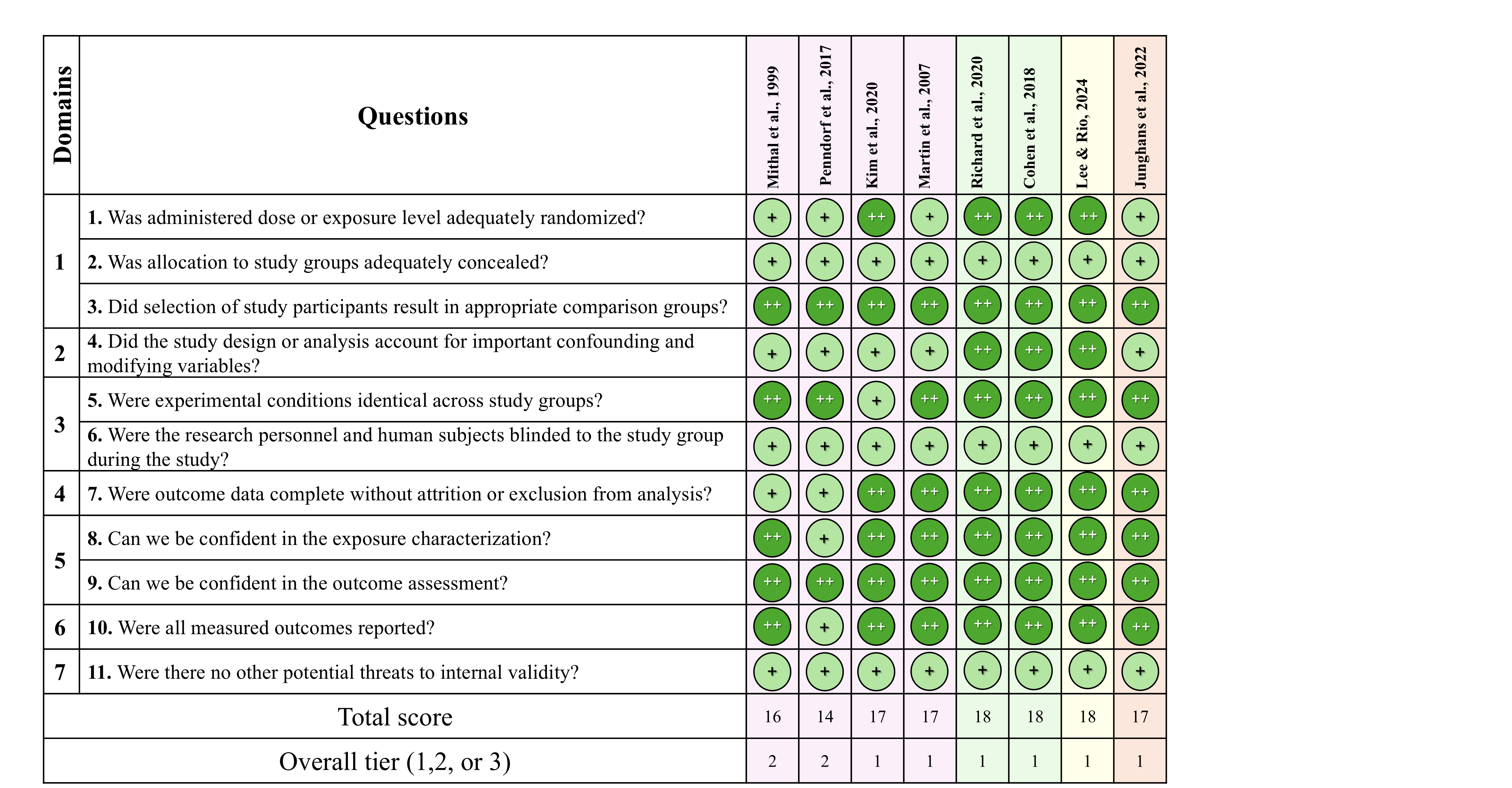

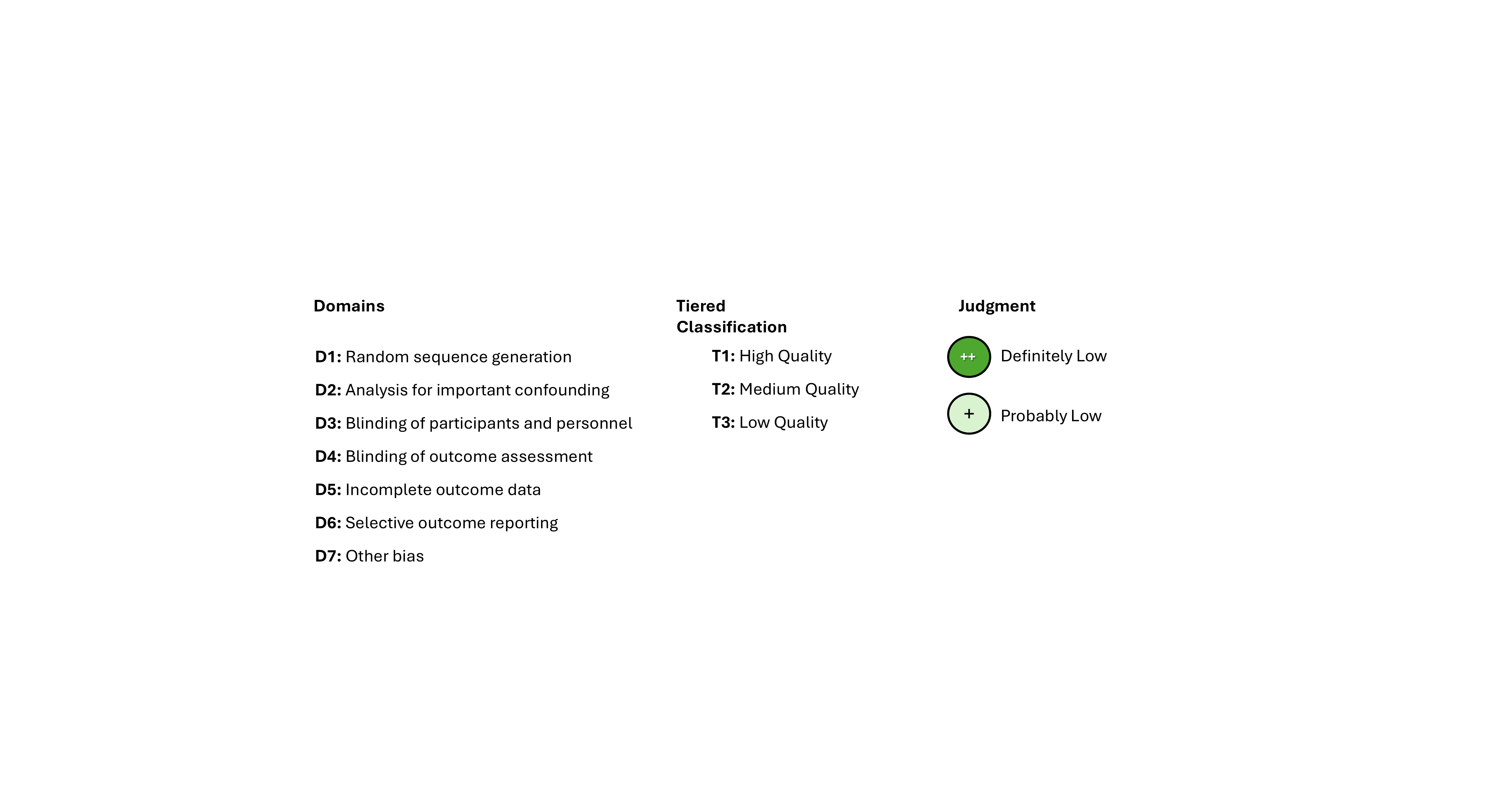


**(A)**

**(B)**

Supplementary Figure 2. OHAT Risk of bias assessment for the eight *in vitro* studies included in the supplementary analysis. (A) Background color indicates the ALS-FTD-associated gene studied: Pink: *SOD1* (4 studies), Green: *SETX* (2 studies), Yellow: *hnRNPA1* (1 study), and Orange: *Vps54* (1 study). Each column corresponds to a study, and each row represents a signaling question related to internal validity domains. Symbols represent risk of bias judgment: “+” = probably low risk, “++” = definitely low risk. (B) Key for domain classification, tiered rating system (T1–T3), and interpretation of judgment scores used in the OHAT assessment.


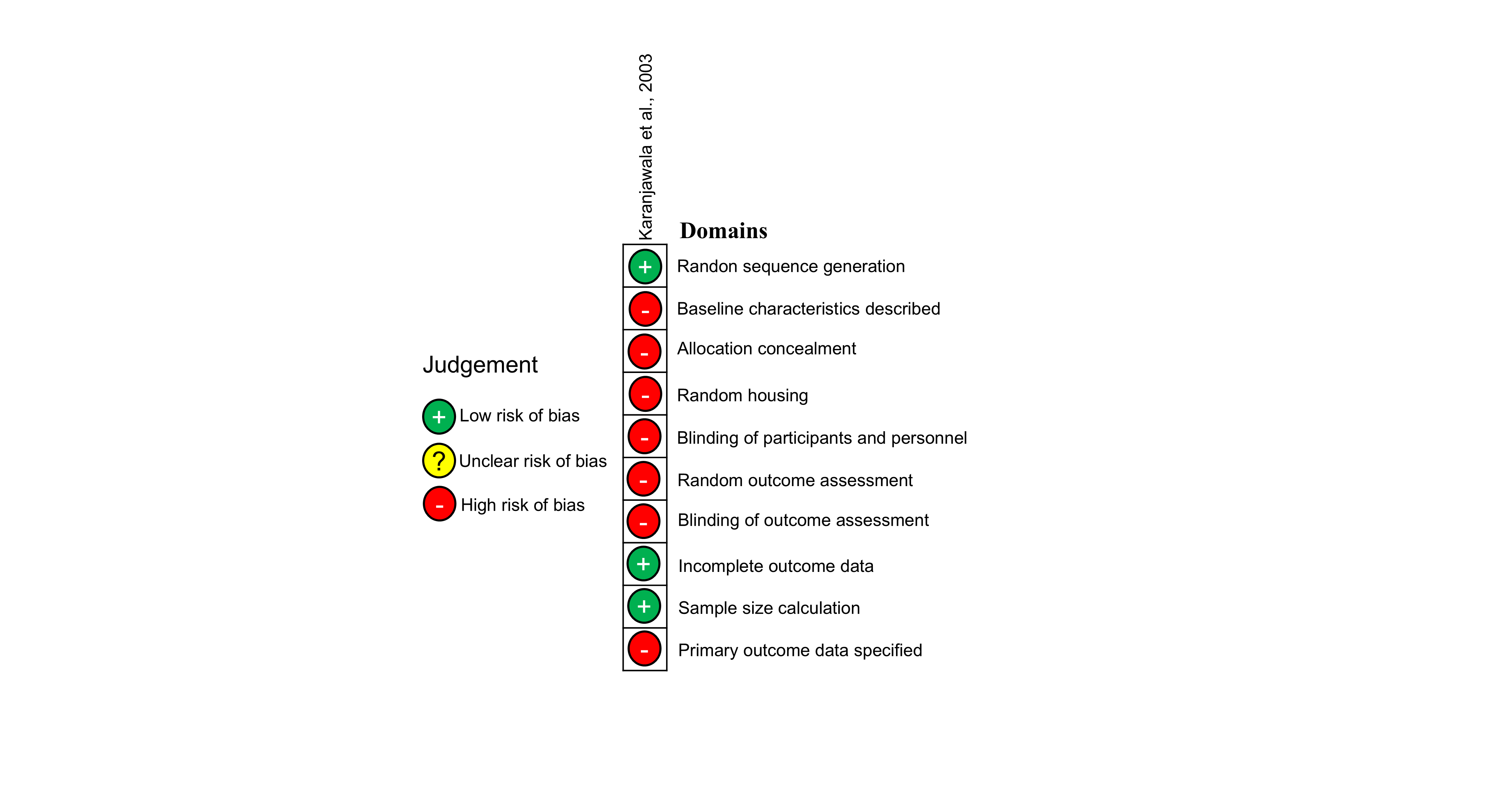


Supplementary Figure 3. SYRCLE Risk of Bias tool for the *in vivo* study by Karanjawala et al. 2003.

## Supplementary Tables

| Study | Country | Model | Intervention | SOD1 Mutation | Key Findings | Methods |  |
| --- | --- | --- | --- | --- | --- | --- | --- |
| **A) Cell line-based studies** | | | | | | | |
| **Penndorf et al., 2017** (8) | Germany | Mouse MN cultures | - | G93A | No evidence of increased DNA damage | γH2AX staining |  |
| **Mithal et al., 1999** (6) | UK | lymphoblastoid from sALS and fALS patients | IR (0-8 Gy) | Ala4Val/ Gly37Arg/Ile149Thr | No significant difference in DSB formation between SOD1 mutant fALS, sALS, and controls | Pulsed-field gel electrophoresis (PFGE) |  |
| **B) iPSC-derived motor neurons from ALS patients** | | | | | | | |
| **Kim et al., 2020** (4) | USA | MN with fALS | ETO (10 μM) | fALS with A4V/ introduction of G93A mutation by CRISPR-Cas9 | No evidence of increased DNA damage even after ETO | γH2AX staining |  |
| **C) Rodent-based studies** | | | | | | | |
| **Penndorf et al., 2017** (8) | Germany | SC tissue (mouse) | - | G93A | No evidence of increased DNA damage | γH2AX staining/ comet assay |  |
| **Martin et al., 2007** (7) | USA | Mouse | - | G93A | Early detection of DNA damage (SSBs) (P<0.01)/ mitochondrial DNA damage detected/ disrupted DDR pathway | TUNEL assay/PANT assay/  IHC/ Stereological and Morphological assessment |  |
| **Karanjawala et al., 2003** (9) | USA | Ku86 KO mice crossed with SOD1 tg mice | - | SOD1 overexpression | In mice deficient in NHEJ due to Ku86 knockout, overexpression of wild-type SOD1 was embryonically lethal in one breeding scheme but not in another,  A genetic modifier on chromosome 13 may explain why some Ku86^−/−^/SOD1 mice survive despite similar SOD1 levels and prenatal lethality in others. | Offspring genotype analysis/ WB/ Genome-wide marker analysis of DNA |  |

**Supplementary Table 1.** Characteristics of the included studies: SOD1

**Supplementary Table 2.** Characteristics of the included studies: Senataxin (SETX)

| Study | Country | Model | Interventions | Research Focus | Key Findings | Methods |
| --- | --- | --- | --- | --- | --- | --- |
| **Human cell line-based studies** | | | | | |  |
| **Richard et al., 2020** (10) | USA | U87 | SETX KD using siRNA | SETX role in autophagy regulation and DNA damage response | SETX involvement in autophagy regulation/ siSETX cells led to upregulation of γH2AX level/ alteration in gene expression | Western blot/ IF/DRIP-seq |
| **Cohen et al., 2018** (11) | France | U20S | SETX KD shRNA/ Etoposide ETO/ IR | SETX role in DNA  double-strand  break repair | SETX showed a preference to be recruited to DSBs in transcriptionally active sites/ increase in DSB-dependent R-loop/ decrease in RAD51 recruitment to DSBs/ an increase in illegitimate rejoining of distant DNA ends | Western blot/ ChIP-seq/ HR, NHEJ, and SSA repair assays |

Supplementary Table 3. Characteristics of the included studies: Heterogeneous nuclear ribonucleoprotein1 hnRNPA1 (hnRNPA1) a known pre-mRNA splicing factor

| Study | Country | Model | Interventions | Research Focus | Key Findings | Methods |
| --- | --- | --- | --- | --- | --- | --- |
| **Human cell line-based studies** | | | | | |  |
| **Lee & Rio, 2024** (12) | USA | FLP-In-293 cells/ SH-SY5Y | CRISPR-Cas9 to insert D262V in endogenous hnRNPA1 | hnRNPA1mutationalters normal RNA-dependent protein–protein interactions | D262V mutation altered the splicing patterns of numerous genes involved in DDR and DNA repair | RNA-seq |

**Supplementary Table 4.** Characteristics of the included studies: *Vps54*

| Study | Country | Model | Interventions | Research Focus | Key Findings | Methods |
| --- | --- | --- | --- | --- | --- | --- |
| **Human cell line-based studies** | | | | | |  |
| **Junghans et al., 2022** (5) | Germany | Wobbler mouse | - | - | Increased DDR factors at both mRNA and protein levels in cervical SC (p < 0.01)/ Increase ROS levels in the MNs correlates with increased abundance of the DDR γH2ax (p < 0.0001)/ Reduced DDR by ROS scavengers | qPCR/WE/ γH2AX staining/ Live cell imaging |
